# Identification of active gaseous-alkane degraders at natural gas seeps

**DOI:** 10.1038/s41396-022-01211-0

**Published:** 2022-03-22

**Authors:** Muhammad Farhan Ul Haque, Marcela Hernández, Andrew T. Crombie, J. Colin Murrell

**Affiliations:** 1 School of Environmental Sciences, University of East Anglia, Norwich, NR4 7TJ UK; 2 School of Biological Sciences, University of the Punjab, Quaid-i-Azam Campus, Lahore, 54000 Pakistan; 3 School of Biological Sciences, University of East Anglia, Norwich, NR4 7TJ UK

**Keywords:** Metagenomics, Environmental microbiology

## Abstract

Natural gas seeps release significant amounts of methane and other gases including ethane and propane contributing to global climate change. In this study, bacterial actively consuming short-chain alkanes were identified by cultivation, whole-genome sequencing, and stable-isotope probing (SIP)-metagenomics using ^13^C-propane and ^13^C-ethane from two different natural gas seeps, Pipe Creek and Andreiasu Everlasting Fire. Nearly 100 metagenome-assembled genomes (MAGs) (completeness 70–99%) were recovered from both sites. Among these, 16 MAGs had genes encoding the soluble di-iron monooxygenase (SDIMO). The MAGs were affiliated to Actinobacteria (two MAGs), Alphaproteobacteria (ten MAGs), and Gammaproteobacteria (four MAGs). Additionally, three gaseous-alkane degraders were isolated in pure culture, all of which could grow on ethane, propane, and butane and possessed SDIMO-related genes. Two *Rhodoblastus* strains (PC2 and PC3) were from Pipe Creek and a *Mycolicibacterium* strain (ANDR5) from Andreiasu. Strains PC2 and PC3 encoded putative butane monooxygenases (MOs) and strain ANDR5 contained a propane MO. *Mycolicibacterium* strain ANDR5 and MAG19a, highly abundant in incubations with ^13^C-ethane, share an amino acid identity (AAI) of 99.3%. We show using a combination of enrichment and isolation, and cultivation-independent techniques, that these natural gas seeps contain a diverse community of active bacteria oxidising gaseous-alkanes, which play an important role in biogeochemical cycling of natural gas.

## Introduction

Approximately 600–900 million tonnes of the potent greenhouse gas methane are released annually into the atmosphere [1] and it has a high global warming potential [2]. The largest source of methane globally is biogenic methane which arises from the activities of methanogenic archaea in anoxic environments [1]. Thermogenic methane of geological origin (often termed “natural gas”) is emitted to the atmosphere as a consequence of anthropogenic fossil fuel extraction, transport and seepage from natural sources, and unintentional release of natural gas due to leaking gas pipelines, coal mining and shale-gas extraction and incidents such as the Deepwater Horizon disaster of 2010 [3]. Natural gas seeps in terrestrial, freshwater, and marine geothermal regions, submarine volcanoes and mud volcanoes, account for the release of ~45 million tonnes of methane per year [1, 4, 5].

Thermogenic natural gas comprises mainly of methane but also contains substantial amounts of other climate-active gases such as ethane and propane. Ethane is a photochemical pollutant while propane is an ozone precursor and together their estimated global emission is around 24–30 million tonnes per annum [6, 7] of which approximately 3–6.4 million tonnes are from natural geologic sources [8]. Some natural gas seeps such as the Eternal Flames in Chestnut Ridge County Park and Pipe Creek, New York State [9–11] release methane containing high concentrations of ethane and propane, and thus provide potential hotspots for microbes that degrade gaseous hydrocarbons.

The major sink for methane and short-chain alkanes once in the atmosphere is photochemical oxidation by hydroxyl radicals [12, 13] but it is estimated that over 50% of methane from both biogenic and thermogenic sources that is released into the biosphere is consumed by methane-oxidising microbes in aerobic and anaerobic environments [14]. Aerobic methane-oxidising bacteria (methanotrophs) [15] grow using either particulate methane monooxygenase (pMMO) or soluble methane monooxygenase (sMMO), a member of the soluble diiron centre (SDIMO) family of oxygenases [16, 17].

Facultative methanotrophs of the genus *Methylocella* can grow simultaneously on both methane and propane using sMMO and another SDIMO enzyme, propane monooxygenase [18]. This metabolic versatility of *Methylocella*, which is widespread in the environment [10, 19–21] stimulated the examination of terrestrial natural gas seep environments rich in both methane and propane.

By comparison with methane, the bacterial metabolism of ethane, propane, and butane is less-well understood and the ecology of bacteria using these gases has not been explored in depth at terrestrial natural gas seeps (the subject of this study). The majority of C_2_–C_4_ alkane-utilising bacteria belong to the order *Actinomycetales* and include members of the genera *Mycolicibacterium* (formerly *Mycobacterium* [22]), *Corynebacterium*, *Nocardia*, and *Pseudonocardia* [23–25], and *Rhodococcus* [26, 27]. Gram-negative C_2_–C_4_ alkane utilisers include *Pseudomonas* [28] and *Thauera* [29] (reviewed by Rojo [30] and Shennan [31]). The initial oxidation of gaseous-alkanes is usually catalysed by an SDIMO enzyme consisting of an oxygenase, a coupling protein, and a reductase. Examination of the evolutionary relationships between different SDIMOs provides a framework for classification of these enzymes into 6 groups containing aromatic/alkene monooxygenases (Group I), phenol hydroxylase (Group II), sMMOs (Group III), and alkane and alkene monooxygenases (Groups IV-VI) [17, 32, 33], although the boundaries of this classification scheme are often blurred. For example, the butane oxidizer *Thauera butanivorans* [34] contains a butane monooxygenase which is more closely related to sMMO (Group III). *Mycolicibacterium chubuense* NBB4, a strain isolated on ethene and growing on C2-C4 alkanes, contains four distinct SDIMOs gene clusters from Groups III and VI, and two from Group IV [25, 35]. It also contains a gene cluster encoding a membrane-bound copper-containing monooxygenase, associated with gaseous-alkane utilisation [36, 37]. *Gordonia* sp. TY-5 oxidises propane via *prmABCD* from Group V [38] which is closely related to the propane monooxygenases (*prmABCD*) of *Methylocella silvestris* BL2 [18], *Rhodococcus* sp. strain BCP1 [27], and *Pseudonocardia* sp. strain TY-7 [24]. Another *Mycobacterium* strain TY-6 contains a *prmACDB* from Group VI [24]. With the exception of *Methylocella*, no C2-C4 alkane utilisers can grow on methane (reviewed in Shennan [31]).

The availability of SDIMO sequences predicted to be involved in gaseous-alkane utilisation, and of extant reference bacteria to investigate their metabolism, has provided a robust taxonomic framework to examine the molecular ecology of these bacteria. DNA sequence retrieval of SDIMOs by PCR [32, 39] or via metagenomics [40–43] can reveal the diversity and abundance of key genes encoding putative gaseous-alkane monooxygenases. Combining DNA-SIP [44, 45], using ^13^C-labelled gases ethane and propane, with a metagenomics analysis of labelled heavy (^13^C) will assist in assigning the relevant SDIMO gene clusters to their function in gaseous-alkane utilisation. The objective of this study was to use this combination of techniques to investigate the presence of active gaseous-alkane degraders at two contrasting natural gas seeps, Pipe Creek [10, 11] and Andreiasu Everlasting Fire [46], previously examined for the presence and activity of *Methylocella* [10, 47]. The hypothesis we were testing was that the analysis of metagenome assembled genomes (MAG) retrieved from ^13^C-labelled DNA would reveal the identity of the bacteria involved. These approaches were combined with cultivation of ethane and propane degraders from these natural gas seeps.

## Material and methods

### Sample collection

Samples from two natural gas seeps sites, Andreiasu (pH 8.2) in Romania and Pipe Creek (pH 6.0) in the USA, were collected for this study. Samples from the Romanian site were taken from the liquid mud through which the thermogenic gas bubbles, as described by Baciu et al. [46]. Pipe Creek (geology described by Schimmelmann et al. [11]) is a fast-flowing stream with a rocky bed. Samples consisted of water and sediment taken from the vicinity of fissures in the stream bed, through which gas was emerging. Varying characteristics of physical nature, pH and the proportion of methane, ethane and propane in the gas emitted from both sites along with sampling conditions have been previously reported [10].

### Enrichment cultures, isolations, and genome sequencing of novel isolates

Fresh samples (~1 g) from natural gas seeps were incubated in 10 ml modified dilute nitrate mineral salts (DNMS) medium [47], supplemented with 5 µM lanthanum, in 120 ml sealed serum vials. A mixture of gases (20%, v/v), comprising of methane (70%, v/v), ethane (10%, v/v), and propane (20%, v/v) was injected as the only supplemental source of C and energy and the vials were incubated in a shaker (150 rpm) at 25 °C for three weeks in the dark. Enrichment cultures were serially diluted and plated onto modified DNMS agar plates (supplemented with 5 µM lanthanum) and incubated, under the same mixture of gases (10%, v/v), in a sealed jar. Colonies appearing on the plates after two weeks of incubation were picked and resuspended in 20 µl of sterile modified DNMS medium. Aliquots of this cell suspension were replica-plated onto two plates of DNMS medium (supplemented with 5 µM lanthanum) and incubated under ethane or propane individually in the headspace (10% v/v) as the only source of carbon and energy. Colonies growing under both conditions were considered as ethane/propane utilisers. Purity of these isolates was achieved by continuously plating of serial dilutions of isolate cultures and confirmed by microscopic observations. After confirmation of purity of the isolates, genomic DNA was extracted using the Wizard Genomic DNA Purification Kit (Promega) according to the manufacturer’s instructions. Genome sequencing of these isolates was performed at MicrobesNG (Birmingham, UK) using HiSeq (Illumina) and Nanopore technology and assembled using SPAdes 3.7 into contigs. Genome sequence annotation, exploration and comparative genomics of the isolates was performed using MicroScope [48] an online platform by GenoScope (France) providing a collection of bioinformatic tools.

### Bacterial strains and growth conditions

Modified DNMS medium supplemented with 5 µM lanthanum (LaCl_3_) in 120 ml serum vials (with 20 ml culture volume) was used for the growth of bacterial strains. Cultures were grown in DNMS medium with ethane or propane (10% v/v) separately as the only carbon and energy source. Optical density of the cultures was measured at 540 nm to monitor the growth of liquid cultures. Concentrations of substrate gases in the cultures were quantified as described by Crombie and Murrell [18].

### DNA-SIP incubations

For DNA-SIP incubations, approximately 2 g of soil/sediment suspensions (1:3 sample and ultra-pure water ratio) were incubated in 120 mL sealed serum vials. Substrate gases (^12^C-ethane, ^12^C-propane, ^13^C-ethane, and ^13^C-propane) were injected separately into the headspace of the vials to a concentration of 1% (v/v). Gas consumption was measured in each vial using gas chromatography [18]. There is a trade-off between leaving the timescale of incubations long enough to ensure enough ^13^C-labelled DNA is obtained to undertake metagenome sequencing and conversely that the length of incubation is not too long, so that as much as possible, cross-feeding of ^13^C-label does not occur. The general guidelines of Neufeld et al. (2007) were followed. All incubations were carried out in duplicate for each substrate and for each time point. Time point 1 samples from DNA-SIP incubations were harvested after they had consumed approximately 100 µmol C per g of fresh sample and for time point 2 samples were harvested after they had consumed approximately 200 µmol C per g of fresh sample as measured by gas chromatography (Fig. S1). Molecular analysis of the time point 2 samples showed that sufficient ^13^C-label was incorporated for a successful SIP experiment, therefore those samples were used for further analysis. After incubations, samples were harvested by centrifugation at 10,000 × *g* for 15 min, supernatants were discarded, and the pellets were stored at −20 °C. DNA was extracted from both native samples without incubations and SIP-incubated samples (~0.5 g) by using the spin kit for soil (MP Biomedicals) following the manufacturer’s instructions. DNA was quantified using a Qubit 2.0 fluorometer (Invitrogen) and by NanoDrop (Thermo Fisher Scientific).

CsCl density gradient ultracentrifugation (177,000 × *g*, 40 h, 20 °C, Beckman Vti 65.2 rotor) and fractionation was used for separating labelled and unlabelled DNA from SIP-incubated samples [41, 49] using the refractive index of CsCl fractions (12 fractions per sample) as a proxy for density. Refractive index of each fraction was measured using a refractometer (Reichert AR200, Reichert Analytical Instruments, Buffalo, USA). The buoyant density of each fraction was calculated as described previously [50] and plotted against the quantity of DNA retrieved from corresponding fraction (Fig. S2). Based on the data shown in Figure S2, three to four fractions of each sample containing labelled DNA (buoyant densities 1.7491–1.7296 g/ml) were mixed and designated as the “heavy” DNA fraction, while two to three fractions of each sample containing unlabelled DNA (buoyant densities 1.7216–1.7123 g/ml) were mixed and designated as the “light” DNA fraction.

### Sequencing

The microbial community of both native and DNA-SIP incubated samples was characterised by sequencing the 16S rRNA gene as previously described [47]. PCR amplicons using universal primers 341 F and 785 R targeting the V3-V4 regions [51] were obtained from the unfractionated unenriched native DNA samples and the heavy and light DNA fractions from DNA-SIP incubated samples. These amplicons were processed as described previously [47] and sequenced using the MiSeq (Illumina) platform of MR DNA (Shallowater, USA).

The total metagenomic DNA of the native samples, plus DNA from heavy fractions from incubations with ^13^C-ethane and ^13^C-propane from both sites (total of six samples) were sequenced on a HiSeq (Illumina) at Novogene (Cambridge, UK). The metagenome was analysed on a high-performance computing cluster supported by the Research and Specialist Computing Support Service at the University of East Anglia (Norwich, UK).

### Bioinformatics

MRDNA proprietary analysis pipeline (www.mrdnalab.com) was used to analyse the sequence data from 16S rRNA gene amplicons as described previously [47]. Operational taxonomic units (OTUs) were defined at a sequence identity level of 97%. Final taxonomic classification of OTUs was performed by BLASTn against databases from RDPII and NCBI using a bootstrap confidence threshold of 97% (www.ncbi.nlm.nih.gov, http://rdp.cme.msu.edu). Taxa fulfilling the following criteria were identified as labelled: (1) the relative abundance in the heavy DNA fraction of the ^13^C-ethane/^13^C-propane-incubated sample was >1.0%, (2) the abundance in the heavy DNA fraction of the ^13^C-ethane/^13^C-propane -incubated sample was higher than the abundance in the light DNA fraction of the ^13^C-ethane/^13^C-propane and (3) the difference in the abundance in the compared heavy and light DNA fractions of the ^13^C-ethane/^13^C-propane-incubated sample was higher than that of the ^12^C-ethane/^12^C-propane-incubated sample.

For metagenomic DNA sequences, reads were checked using FastQC version 0.11.8 [52]. Low-quality reads were discarded using BBDuk version 38.68 [53]. After quality checking, reads were merged into longer contiguous sequences (scaffolds) using de novo assemblers SPAdes (single assembly) and metaSPAdes (co-assembly) version 3.13.1 [54, 55]. Co-assembly was performed for each site (Pipe Creek and Andreiasu) independently. Downstream binning analysis was performed using scaffolds larger than 1000 bp. Metagenomic binning of the assembled scaffolds was carried out with the metaWRAP version 1.2.1 pipeline [56]. Completion and contamination metrics of the extracted bins were estimated using CheckM [57]. The resulting bins were collectively processed to produce consolidated metagenome-assembled genomes (MAGs) using the bin_refinement module (criterion: completeness >70%; contamination <5%). MAGs retrieved from both single- and co-assembly were then dereplicated using dRep [58]. Only the highest scoring MAG from each secondary cluster was retained in the dereplicated set. The abundance of each MAG and genome in the different sites was calculated separately using BLASTn version 2.5.0+ [59, 60], keeping only hits with >95% identity and e-value cutoff <1e^−5^ for the analysis. A final heatmap of the abundance of the isolates and MAGs, containing genes encoding SDIMO and membrane-bound methane monooxygenase (PmoA), was constructed as described previously [60].

### Functional annotation

All the MAGs retrieved in this study and the three isolate genomes were analysed using BLAST separately against databases containing SDIMO-related genes (*mmoX*, *bmoX*, *prmA*, *dmpN*, *tmoA*) and *pmoA* genes. Afterwards, all the genomes of the isolates and only the MAGs that presented hits from the BLAST were annotated using MicroScope platform [48] for downstream analysis. Automatic annotations were validated manually using BLASTp for translated sequences of the genes involved in metabolic pathways of interest, such as those involved in SDIMO (methane monooxygenase, putative butane monooxygenase, propane monooxygenase, toluene monooxygenase and phenol hydroxylase), and membrane-bound methane monooxygenase.

### Phylogenomic analysis

For identification of the isolates, the 16S rRNA gene regions of strains PC2, PC3, and ANDR5 were extracted using Barrnap v. 0.9 software (https://github.com/tseemann/barrnap) and compared with the type strains using BLASTn. Amino acid comparisons between the genomes and MAGs retrieved in this study and their closest-relative type strains were calculated based on reciprocal best hits using the Enveomics collection [61]. Average nucleotide identity (ANI) was calculated using the Enveomics platform and confirmed with the outputs from automated multi-locus species tree analysis (autoMLST) [62]. The recommended species cut-off was 95% for ANI [63] and ~70% for amino acid identity (AAI) indices [64] (Ramon Rosselló-Móra, *pers. comm.)*. In addition, JSpeciesWS software (http://jspecies.ribohost.com/jspeciesws/ [65]) was used to determine the correlation indexes of the tetra-nucleotide signatures between the isolates and their closest type strains.

Genomes of isolates and the MAGs were submitted to the TYGS (Type Strain Genome Server) platform (https://tygs.dsmz.de [66]). This platform predicts digital DNA:DNA hybridisation (dDDH) values of the isolates to the most closely related type strains. Additionally, the translated sequences of a set of ten house-keeping genes (*rpoB, secA, gyrB, rho, murB, era, recR, dapD, aroC*, and *nusG*), as well as SDIMO genes (i.e. strains PC2 and PC3 (*mmoX*), and strain ADNR5 (*prmA*)) were retrieved from both the isolates and their closest type strains for comparisons using BLASTx.

The taxonomic classification of MAGs was performed using the classify_bins module from metaWRAP which relies on the NCBI_nt database. A phylogenomic tree, which included both isolates and MAGs was created using the autoMLST pipeline and conducted with 1000 bootstrap replicates [62]. Phylogenetic analysis of the key SDIMO and membrane-bound methane monooxygenase enzymes was conducted in MEGA X [67] using the Maximum Likelihood method with a JTT matrix-based model and 100 bootstrap replicates.

## Results and discussion

Samples from Pipe Creek [11] and Andreiasu [46] were examined for the presence and activity of gaseous-alkane degrading bacteria using cultivation-independent techniques. These sites were chosen because the natural gas emitted at these sites had been previously shown to contain substantial amounts of ethane and propane, as well as methane, and were known to harbour *Methylocella* that could use methane, ethane, and propane [10, 47]. The goal of this study was to determine which ethane- and propane-utilising bacteria were active at these sites and to investigate the soluble di-iron centre monooxygenases, which enable them to grow on gaseous-alkanes.

### Incubation of natural gas seep samples with ethane and propane

**Fig. 1:**
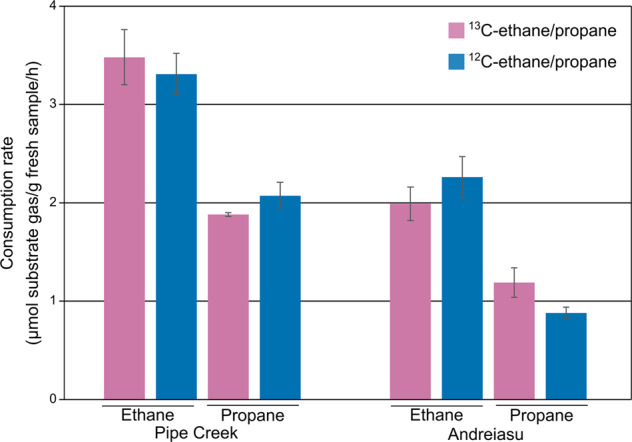
Consumption rates of propane and ethane by environmental samples from natural gas seep sites. Bars represent mean values with standard deviations of independent duplicate incubations for each substrate.

To confirm that samples from natural gas seeps were active and to examine the bacterial community consuming ethane and propane, Pipe Creek and Andreiasu samples were incubated with ^12^C- or ^13^C-labelled ethane or propane. There were no major differences in the consumption of ^13^C-substrate gases compared to ^12^C-substrate gases in samples from Pipe Creek or Andreiasu. However, Pipe Creek samples consumed ethane and propane faster than samples from Andreiasu (Fig. 1, Fig. S1). This is supported by the fact that the natural gas emitted from Pipe Creek seep site contained very high amounts (~5–10x higher) of ethane and propane compared to those present in natural gas emitted from Andreiasu site [10, 11, 46]. This also suggests that potential ethane and propane consumers at Pipe Creek may have been more abundant and better adapted to ethane and propane utilisation as reported previously [47]. These bacteria may serve as a natural biofilter for gases before they are emitted to the atmosphere.

SIP was performed in order to examine the diversity of active ethane- and propane-degrading bacteria at both natural gas seep sites. DNA from incubations with both ^12^C- and ^13^C-ethane or propane was separated by isopycnic density gradient centrifugation to yield light and heavy DNA fractions (Fig. S2). This yielded a total of 16 DNA samples: (a) light and heavy DNA from Pipe Creek samples incubated with ^12^C-ethane or ^13^C-ethane, and three more corresponding sets of four samples from (b) Pipe Creek incubations with ^12^C-propane or ^13^C-propane; (c) Andreiasu incubations with ^12^C-ethane or ^13^C-ethane; (d) Andreiasu incubations with ^12^C-propane or ^13^C-propane.

### 16S rRNA genes of putative ethane- and propane-utilising bacteria

Profiles of the relative abundance of 16S rRNA genes in the light and heavy DNA fractions from incubations of Pipe Creek samples with ^12^C-ethane were similar and as expected for a successful DNA-SIP experiment (Fig. S3A). A background of unlabelled DNA is usually present in the heavy fractions in all SIP experiments (Fig. S2). However, heavy fractions were always enriched in labelled DNA compared with the controls (Fig. S2). Focussing on the heavy DNA, the relative abundance of 16S rRNA genes labelled with ^13^C-ethane revealed that active members of the phylum Alphaproteobacteria including the genera *Sphingomonas* (having the highest relative abundance of ~27%), *Rhodobacter*, *Xanthobacter *and a genus belonging to Gammaproteobacteria (*Hydrogenophaga*, formerly Betaproteobacteria) were the most abundant in Pipe Creek (Fig. S3A). The most abundant putative ethane-degrading bacteria labelled in Andreiasu samples belonged to the genera *Mycolicibacterium* (Actinobacteria, 32%) and *Micavibrio* (Alphaproteobacteria, 18%) (Fig. S3B). Even though high abundances were observed with members of *Sphingomonas* and *Micavibrio*, no MAGs containing SDIMO genes from these genera were retrieved after SIP incubations.

The relative abundance of 16S rRNA genes in heavy DNA from incubations with ^13^C-labelled propane was analysed for both sites. For Pipe Creek samples, the genus with the highest relative abundance was *Hydrogenophaga* (13%) together with four other abundant genera from the class Alphaproteobacteria (*Xanthobacter*, *Sphingobium, Sphingomonas* and *Rhodobacter*, ~7–8%) (Fig. S4A). Interestingly, these genera featured as some of the most abundant in ^13^C-ethane DNA-SIP experiments with Pipe Creek samples (Fig. S3A). From Andreiasu samples, the most abundant genera in incubations with ^13^C-propane were *Mycolicibacterium* (19%), *Hydrogenophaga* (9%), as well as *Rhizobium* and *Rhodobacter* (both ~5%) (Fig. S4B). Some of these strains contain monooxygenases that are known or predicted to be involved in gaseous hydrocarbon metabolism (e.g. *Hydrogenophaga* sp. T4 [43], *Xanthobacter* sp. Py2 [68] and *Sphingobium* [19]).

### Isolation and genome sequencing of gaseous-alkane degraders

**Fig. 2:**
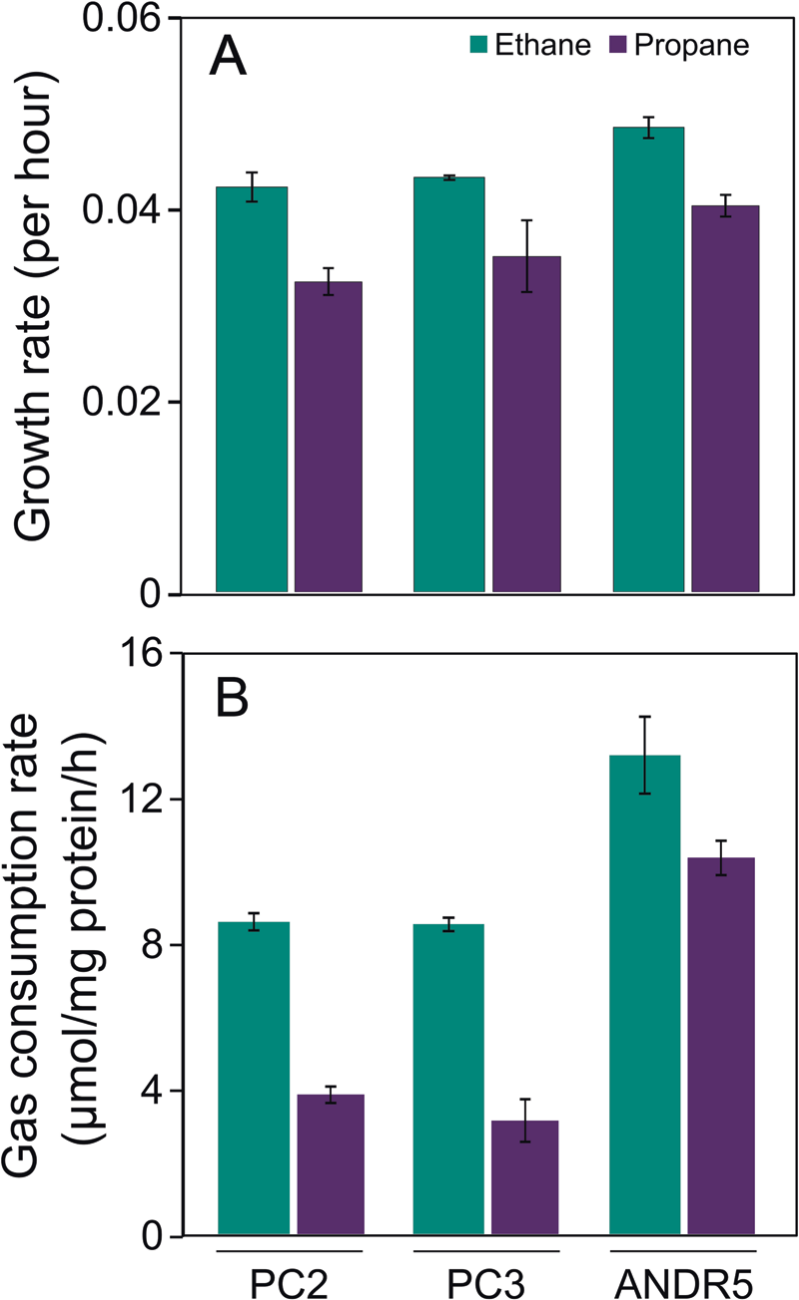
Growth and substrate consumption of the isolates. Growth rates (**A**) and gas consumption (**B**) of strains PC2, PC3, and ANDR5. Bars represent mean values with standard deviations of independent duplicate incubations for each substrate.

The identification by DNA-SIP and screening of 16S rRNA genes from ^13^C-DNA samples of putative ethane and propane degraders prompted us to attempt to enrich and isolate key members of the gaseous alkane-degrading community at these sites. Two new isolates were obtained from Pipe Creek (PC2 and PC3) and one from Andreiasu (ANDR5). All isolates grew well on ethane and propane (but not on methane) (Fig. 2A, Fig. S5) and could consume approximately 10% (v/v) of these gaseous-alkanes in the headspace of cultures within 200 h (Fig. 2B, Fig. S5). The completeness of these genomes ranged from 97.9–99.8% and contamination was <5%. The 16S rRNA gene sequences of isolates PC2 and PC3 were highly similar to *Rhodoblastus acidophilus* DSM 137 ^T^ (97.6%, PRJNA396223), and isolate ANDR5 was highly similar to *Mycolicibacterium litorale* F4^T^ (97.6%, PRJNA374925) (Table S1). The genomes of PC2 and PC3 reveal an AAI of 79% to their closest relative, type strain *Rhodoblastus acidophilus* (formerly *Rhodopseudomonas acidophila* [69]) and the genome of ANDR5 indicates an AAI of 82% to *Mycolicibacterium litorale* [70] (Table 1).

**Table 1:** Summary of metagenome-assembled genomes and whole genome sequencing (WGS).

ID	Origin	MAG retrieved from	Material	Completeness	Contamination	Length	Identification	Reference type strain autoMLST (GenBank accession number)	AAI	ANI
				(%)	(%)	(bp)			(%)	(%)
MAG12a	Andreaisu	single assembly - ethane incubations	DNA-seq	86.9	4.8	2729405	*Methylovulum*	*Methylovulum miyakonense* HT12 (GCF_000384075)	69.7	76.2
MAG19a	Andreaisu	co-assembly	DNA-seq	99.4	1.3	3815928	*Mycolicibacterium*	*Mycolicibacterium litorale* F4 (GCA_002007745)	79.5	82.7
MAG22a	Andreaisu	single assembly - propane incubations	DNA-seq	78.4	3.8	3341519	*Dechloromonas*	*Dechloromonas denitrificans* ATCC BAA-841 (GCF_001551835)	78.2	82.6
MAG26a	Andreaisu	single assembly - ethane incubations	DNA-seq	98.9	0.9	4339169	*Hydrogenophaga*	*Hydrogenophaga pseudoflava* NBRC 102511 (GCF_001592285)	77.4	85.4
MAG27a	Andreaisu	single assembly - propane incubations	DNA-seq	78.5	4.1	3487664	*Hydrogenophaga*	*Hydrogenophaga palleronii* NBRC 102513 (GCF_001571225)	77.2	83.8
MAG32a	Andreaisu	co-assembly	DNA-seq	97.1	1.4	3163398	Rhizobiales	*Rhodomicrobium vannielii* ATCC 17100 (GCF_000166055)	62.0	72.6
MAG33a	Andreaisu	single assembly - ethane incubations	DNA-seq	82.5	1.3	4651566	Rhizobiales	*Starkeya novella* DSM 506 (GCF_000092925)	52.9	75.5
MAG2p	Pipe Creek	co-assembly	DNA-seq	89.8	1.9	4093978	*Mycolicibacterium*	*Mycolicibacterium vanbaalenii* PYR-1 (GCF_000015305)	70.6	78.3
MAG14p^a^	Pipe Creek	single assembly-original sample	DNA-seq	98.7	0.2	3486745	*Methylocapsa*	*Methylocapsa aurea* KYG (GCF_000746085)	77.4	81.5
MAG15p	Pipe Creek	single assembly - ethane incubations	DNA-seq	85.9	2.2	2934464	*Rhodoblastus*	*Rhodoblastus acidophilus* DSM 137 (GCF_900187365)	68.8	80.8
MAG16p	Pipe Creek	single assembly - ethane incubations	DNA-seq	94.9	2.2	3860394	*Rhodoblastus*	*Rhodoblastus acidophilus* DSM 137 (GCF_900187365)	68.6	78.6
MAG17p	Pipe Creek	single assembly - ethane incubations	DNA-seq	82.4	0.5	2824381	*Methylocella*	*Methylocella silvestris* BL2 (GCF_000021745)	73.7	80.7
MAG18p	Pipe Creek	co-assembly	DNA-seq	96.7	0.9	3990415	*Methylocella*	*Methylocella silvestris* BL2 (GCF_000021745)	74.9	80.2
MAG38p	Pipe Creek	co-assembly	DNA-seq	87.7	2.4	3944496	*Rhodoferax*	*Rhodoferax fermentans* JCM 7819 (GCF_002017865)	79.6	81.6
MAG39p	Pipe Creek	single assembly - ethane incubations	DNA-seq	91.0	2.7	3229017	*Xanthobacter*	*Xanthobacter autotrophicus* Py2 (GCF_000017645)	74.7	84.6
MAG47p	Pipe Creek	co-assembly	DNA-seq	95.7	1.1	3555802	Rhodobacteraceae	*Loktanella pyoseonensis* DSM 21424 (GCF_900102015)	56.2	77.2
MAG49p	Pipe Creek	co-assembly	DNA-seq	70.7	0.0	3567447	Rhodobacteraceae	*Loktanella pyoseonensis* DSM 21424 (GCF_900102015)	58.7	79.0
PC2	Pipe Creek	n.a.	WGS	98.3	0.8	4345403	*Rhodoblastus*	*Rhodoblastus acidophilus* DSM 137 (GCF_900187365)	68.6	79.3
PC3	Pipe Creek	n.a.	WGS	97.9	1.7	4316956	*Rhodoblastus*	*Rhodoblastus acidophilus* DSM 137 (GCF_900187365)	68.4	79.3
ANDR5	Andreaisu	n.a.	WGS	99.8	4.1	4050246	*Mycolicibacterium*	*Mycolicibacterium litorale* F4 (GCA_002007745)	79.5	82.6

*AAI* Amino acid identity (Enveomics), *ANI* Average nucleotide identity (autoMLST), *n.a.* Not applicable.

^a^MAG contains genes encoding pMMO only.

**Fig. 3:**
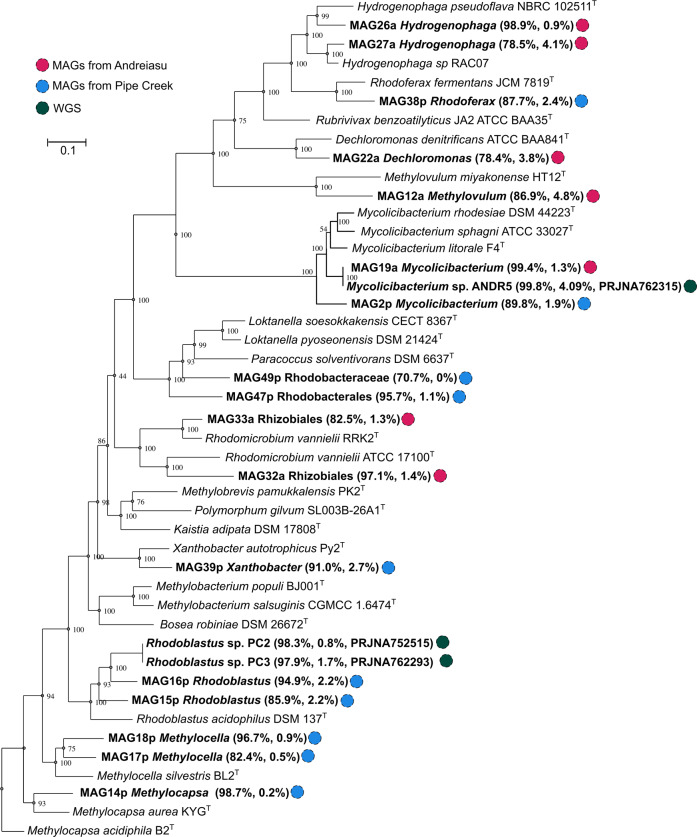
Multi-locus phylogenetic tree of MAGs and strains PC2, PC3, and ANDR5 using autoMLST. Bootstrap confidence levels are indicated at internodes. Ten conserved housekeeping genes (*infC, hemH, yajC, lipA, frr, nusB, fpg, era, truB* and PF00380.15 ribosomal protein S9/S16) were used for the analyses. MAGs and isolates are indicated in bold together with their respective completeness, contamination, and accession number (for isolates). The scale bar represents 10% sequence divergence.

Isolates PC2 and PC3, and two MAGs (MAG15p and MAG16p), all grouped within *Rhodoblastus acidophilus* (Fig. 3). The AAIs among the isolates and these MAGs are 79.4–80.8%, indicating that the MAGs and the isolates are different species. Isolates PC2 and PC3 have a dDDH of 99.9% and AAI of 99.9% which indicates that they are probably the same strain of *R. acidophilus*. Interestingly the genome of *Mycolicibacterium* strain ANDR5 is closely related to MAG19a (Table 1). The multi-locus phylogeny with the genomes of ANDR5 and MAG19a is virtually identical (Fig. 3).

**Fig. 4:**
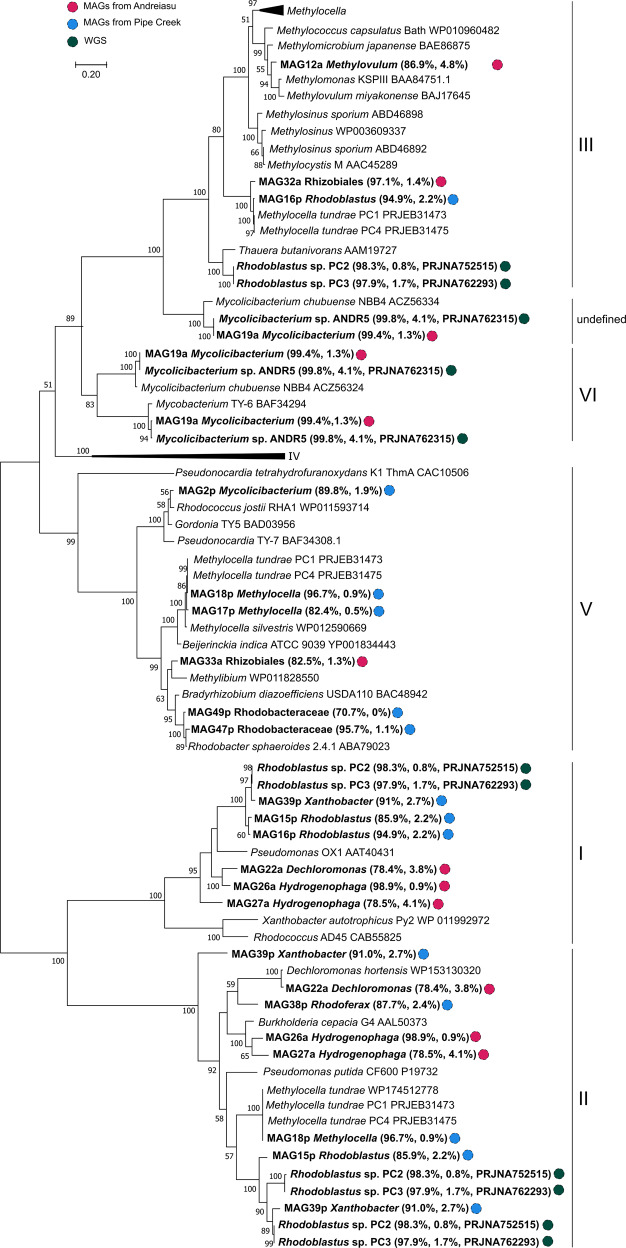
Phylogenetic tree of the SDIMO large subunit proteins using the Maximum Likelihood method with a JTT matrix-based model. Bootstrap values (>50) are shown at the nodes. MAGs and isolates are indicated in bold together with their respective completeness, contamination, and accession number (for isolates). The scale bar represents 20% sequence divergence. SDIMO groups analysed in this study: Group-I: toluene, Group-II: phenol, Group-III: methane and butane, Group-IV: ethene, propene, Group-V and Group-VI: propane [17].

**Fig. 5:**
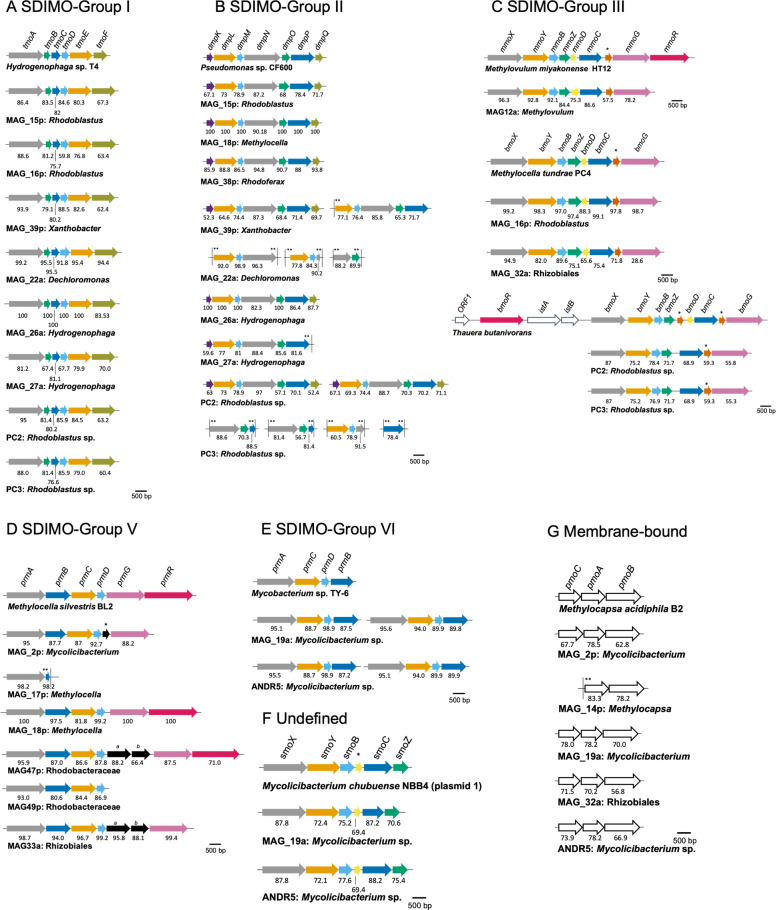
Gene layout of the monooxygenase operons encoded on the MAGs and isolates. Gene clusters of SDIMO (**A**–**F**) and membrane-bound pMMO (**G**). Names of the genes are given above the reference strain. AAIs with homologous proteins of their closest relative (shown in Table S3) are shown below the arrows. *, unknown function (hypothetical protein); **, end of contig; a, amidohydrolase; b, iron-sulfur cluster assembly protein. The descriptions of SDIMO groups are shown in legend of Fig. 4. Colours are indicated as: large (alpha) subunit (grey), small (beta) subunit (orange), gamma subunit (green), coupling protein (light-blue), unknown function (brown), reductase (blue), ferredoxin (khaki), chaperone (pink), transcriptional regulator (dark pink), and auxiliary protein (purple).

The organisation of the SDIMO gene clusters from PC2, PC3, and ANDR5 was also analysed (Fig. 4). Isolates PC2 and PC3 contained a Group I SDIMO gene cluster (nomenclature of Holmes and Coleman, [17]), which includes toluene-o-xylene monooxygenase from *Pseudomonas stutzeri* OX1 [71] and the alkene monooxygenase of *Xanthobacter* Py2 [68] (Fig. 5A), an SDIMO gene cluster of Group II (Fig. 5B), and an SDIMO gene cluster of Group III (Fig. 5C), related to butane monooxygenase of *Thauera butanivorans* [29, 34]. The putative butane monooxygenase of both isolates PC2 and PC3 did not show any “hits” when searched by BLAST against the genome of the type strain of *R. acidophilus* DSM 137 ^T^ (Table S1).

Five MAGs from Pipe Creek and one MAG from Andreiasu contain genes encoding a propane monooxygenase of Group V, but no genes encoding an enzyme from this group were found in the isolates (Fig. 5D). Nevertheless, the isolate *Mycolicibacterium* strain ANDR5 contains an SDIMO gene cluster of Group VI (also encoding a propane monooxygenase) (Fig. 5E), which was very similar to the corresponding SDIMO from MAG19a (Fig. 4) and most closely related to the propane monooxygenase gene cluster of *Mycobacterium* sp. TY-6 [24]. The type strain *M. litorale* F4^T^ only showed 39.9% similarity by BLAST analysis against the propane monooxygenase of isolate ANDR5 (Table S1). Isolate ANDR5 also contains a second undefined SDIMO gene cluster which was again very similar to another SDIMO gene cluster identified in MAG19a (Figs. 4, 5F, Table S2), and which had a high degree of similarity with the SDIMO gene cluster of *Mycolicibacterium chubuense* NBB4 [25]. Strain ANDR5 also contained a gene cluster, designated *pmoCAB*, encoding a putative membrane-bound monooxygenase that was also observed in MAG19a (Fig. 5G, Fig. S6). All of this indicates that the MAG19a genome corresponds to a strain related to ANDR5.

### Analysis of the metagenomes retrieved from DNA-SIP incubations with ^13^C-labelled ethane and propane

Since conventional enrichment and isolation only yielded three representative strains of ethane- and propane-utilising bacteria from these natural gas seep samples, the metagenomes of bacteria labelled in SIP incubations with ^13^C-ethane and ^13^C-propane were examined. For this, the total metagenomic DNA of the original samples, plus DNA from heavy fractions of incubations with ^13^C-ethane or ^13^C-propane from both sites, which is very likely to contain genomes of active hydrocarbon degrading bacteria, were sequenced individually using high-throughput sequencing. In total, 99 MAGs with a completeness >70%, and with a contamination <5% were recovered. 60 of these MAGs originated from Pipe Creek DNA-SIP incubations and 39 MAGs originated from Andreiasu DNA-SIP incubations, and their original samples. Since we aimed to investigate the genomes of putative ethane- and propane-degrading bacteria from both natural gas seep environments, only those MAGs containing gene clusters that resembled either SDIMO gene clusters or membrane-bound monooxygenase gene clusters were selected for detailed analysis. The 17 MAGs selected on the basis of containing putative gaseous hydrocarbon-degrading oxygenases, plus the three genome sequences from the ethane- and propane-degrading isolates, *Rhodoblastus* strains PC2 and PC3, and *Mycolicibacterium* strain ANDR5 described above, were further annotated as described in Methods and summarised in Table 1.

For Pipe Creek, one MAG (MAG2p) was affiliated to Actinobacteria (*Mycolicibacterium*), eight MAGs (MAG14p, MAG15p, MAG16p, MAG17p, MAG18p, MAG39p, MAG47p and MAG49p) belonged to the class Alphaproteobacteria (*Methylocella*, *Rhodoblastus*, *Methylocapsa*, Rhodobacteraceae and *Xanthobacter*), and one MAG (MAG38p) was affiliated to Gammaproteobacteria (*Rhodoferax*) (Table 1). MAG17p and MAG18p, affiliated with *Methylocella*, are interesting because *Methylocella* is an abundant and active facultative methanotroph in Pipe Creek natural gas seeps [10, 47] and since *Methylocella* can grow on ethane and propane [18], it is likely that these could have been enriched in ethane and propane SIP incubations.

**Fig. 6:**
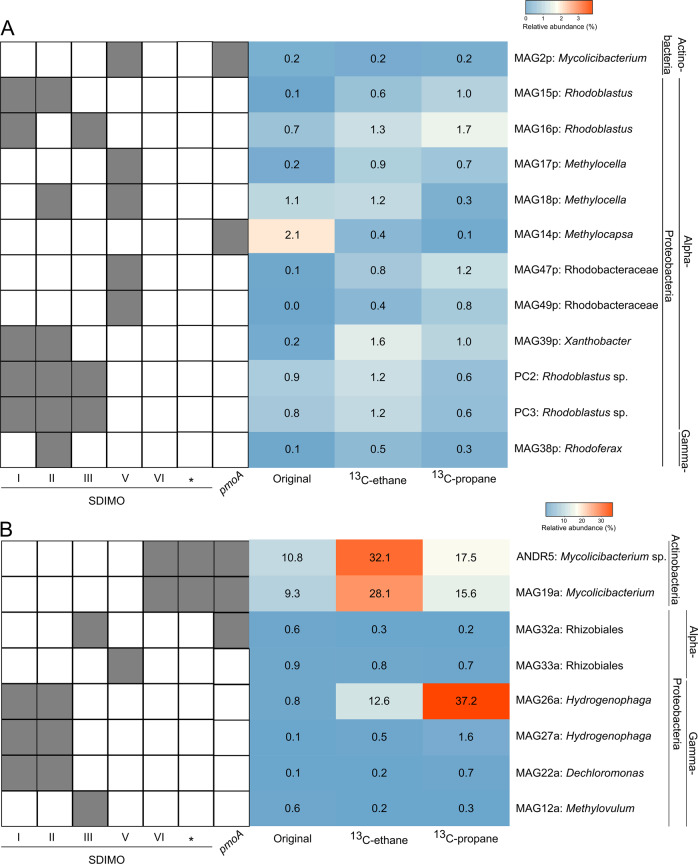
Summary of the presence/absence of SDIMO and membrane-bound MMO gene clusters in each MAG and genome (left panel) and heatmap showing the abundance of those MAGs and isolates in each metagenome (right panel). **A** Pipe Creek; (**B**) Andreiasu. * undefined SDIMO gene cluster. Numbers indicate the abundance of each MAG and genome in the different sites (the analysis was calculated separately using BLASTn, retaining only hits with >95% identity and e-value <1e-5).

For Andreiasu, one MAG (MAG19a) belonged to Actinobacteria (*Mycolicibacterium*), two MAGs (MAG32a and MAG33a) were affiliated to Alphaproteobacteria (Rhizobiales), four MAGs were affiliated to Gammaproteobacteria (MAG22a belonged to *Dechloromonas*, MAGs 26a and 27a belonged to *Hydrogenophaga*, and MAG12a belonged to *Methylovulum*) (Table 1). Of particular note is the estimated high abundance of MAG19a (*Mycolicibacterium*) in DNA labelled with ^13^C-ethane and MAG26a (putative *Hydrogenophaga*) found in DNA labelled with ^13^C-propane (Fig. 6B). The gaseous alkane-degrading isolate *Mycolicibacterium* strain ANDR5 and MAG19a representing a *Mycolicibacterium* strain from Andreiasu samples have a very high AAI of 99.3% suggesting that we have isolated one of the major gaseous-alkane degraders from this natural gas seep site.

### Characterisation of SDIMO and membrane-bound copper-containing monooxygenases in MAGs and the genomes of isolated ethane/propane-degrading bacteria

Representative SDIMO gene clusters in MAGs and isolates were found for all groups of SDIMOs except Group IV SDIMOs [17] (Figs. 4, 5A–E, 6, Table S3). In addition, two near identical undefined SDIMO gene clusters (Table S2), found in MAG19a and *Mycolicibacterium* strain ANDR5 (Fig. 5F), did not cluster within a known SDIMO group. MAG19a and strain ANDR5 also contained a putative pMMO-like alkane monooxygenase (*pmoCAB*, Fig. 5G, Fig. S6), potentially involved in alkane metabolism [36, 37, 43, 72–76]. Also, the individual genes of the *pmoCAB* operon have >99% identity between MAG19a and ANDR5 (Table S2). The phylogeny of these pMMO-like, membrane-bound monooxygenases recovered from MAGs and in *Mycolicibacterium* strain ANDR5 was also analysed. MAG14p (*Methylocapsa*) PmoA is not surprisingly affiliated with PmoA of other methanotrophs (Fig. S6). The PmoA-like polypeptide sequences derived from *Mycolicibacterium* MAGs19a and 2p cluster with the corresponding polypeptide found in *Mycolicibacterium* strain ANDR5 (Fig. S6). Indeed, the PmoA-like polypeptides from strain ANDR5 and MAG19a are virtually identical, again confirming that the isolated ethane/propane degrader from Andreiasu is very representative of a prominent member of the enriched community, retrieved as MAG19a.

Even though their genomes are not identical, both MAGs identified as *Rhodoblastus* (MAG15p and MAG16p) were grouped in the same cluster as the isolates PC2 and PC3 (Figs. 3, 4). MAG15p and MAG16p, as well as isolates PC2 and PC3, contain genes encoding a toluene monooxygenase from Group I SDIMOs (Figs. 4, 5A). Also, MAG15p and MAG16p, and the two isolates contain genes encoding a phenol hydroxylase (Group II SDIMOs, Fig. 5B), although *dmpN* (encoding phenol hydroxylase) in MAG16p was truncated (and therefore not used to build the phylogenetic SDIMO tree). Furthermore, MAG16p and MAG32a contain genes encoding sMMO (Group III SDIMOs, Fig. 5C). No SDIMO gene clusters encoding putative isoprene monooxygenases were detected.

*Rhodoblastus* isolates PC2 and PC3 contained *bmoXYBZDCG*, encoding a putative butane monooxygenase from Group III SDIMOs (Fig. 5C). Strain ANDR5 also grows on butane to an optical density of >1.0, but no genes encoding butane monooxygenase were found. Strains PC2 and PC3 also contained putative toluene monooxygenase (*tmoABCDEF*) and phenol hydroxylase (*dmpKLMNOP*) gene clusters (two copies each) (Fig. 5A, B). *Rhodoblastus* genus was observed in heavy DNA fractions of ^13^C-ethane incubations in Pipe Creek samples (Fig. S3A) but only at low abundance (~1%), therefore we assume that this was not a major player in ethane metabolism at Pipe Creek but nevertheless was one of the easiest alkane degraders to isolate.

An estimation of the abundance of MAGs and isolates containing SDIMO and membrane-bound monooxygenases in DNA from both native and enriched samples was carried out (Fig. 6). The MAG with the highest abundance in the original soil from Pipe Creek site was from *Methylocapsa* (MAG14p, 2.1%). After incubations with ^13^C-labelled ethane, MAGs mostly from *Methylocella* (MAG18p, 1.1%), a MAG also from the class Rhizobiales (MAG16p, 1.3%) and *Xanthobacter* (MAG39p, 1.6%) became enriched. Also, the genomes of *Rhodoblastus* strains PC2 and PC3 had higher abundance within the metagenomes retrieved from incubations with ^13^C-labelled ethane than with ^13^C-labelled propane. After incubations with propane, several alphaproteobacterial MAGs were highly abundant (MAG16p: 1.7%, MAG39p: 1.0%, and MAG47p: 1.2%, Fig. 6A). The MAG with the highest abundance retrieved after incubating Andreiasu samples with labelled ethane belonged to the phylum Actinobacteria (MAG19a, 28.1%). Also, the isolate *Mycolicibacterium* strain ANDR5 was highly abundant in the raw metagenome dataset from the SIP incubations with ^13^C-ethane (32.1%). After incubations with ^13^C-labelled propane, the MAG with the highest abundance belonged to *Hydrogenophaga* (MAG26a, 37.2%), which was also abundant (12.6%) after incubation with ethane (Fig. 6B).

### Description of *Candidatus* “Mycolicibacterium alkanivorans” sp. nov. and *Candidatus* “Rhodoblastus alkanivorans” sp. nov

Further genomic comparisons of the three isolates using TETRA, dDDH, ANI, AAI, and a set of ten house-keeping genes, revealed similarities below species-level thresholds when the type strain genome sequences were used as query (Table S1). This indicates that these three isolates are novel. Strain ANDR5 is the representative of a novel species of the genus *Mycolicibacterium* within the order *Corynebacteriales* (Phylum *Actinobacteria*). Strains PC2 and PC3 are novel species of the genus *Rhodoblastus* within the order *Rhizobiales* (Phylum *Proteobacteria*). Since both strains PC2 and PC3 shared an ANI of 99.99% between each other, we are proposing PC2 genome as the type material for this candidate new species. All isolates grew well on ethane, propane, and butane as carbon sources, and possessed SDIMO-related genes. We propose the following new *Candidatus* species:

### Description of *Candidatus* “Mycolicibacterium alkanivorans” sp.nov

*Candidatus* M. alkanivorans (al.ka.ni.vo’rans. N.L. neut. n. *alkanum*, saturated aliphatic hydrocarbon; L. inf. v. *vorare*, to eat; N.L. part. adj. *alkanivorans*, alkane-devouring).

Gram-positive rod-shaped aerobic bacterium isolated from natural gas seep (liquid mud) located in Romania. The isolate ANDR5 contains SDIMO-related genes including a propane monooxygenase. The isolate ANDR5 also contained a gene cluster encoding a putative membrane-bound monooxygenase. The genome is characterised by a size of 4.3 Mb and has a G + C content of 63.7 mol%.

The type genome of the species has been deposited at the NCBI under the accession number PRJNA762315.

### Description of *Candidatus* “Rhodoblastus alkanivorans” sp.nov

*Candidatus* R. alkanivorans (al.ka.ni.vo’rans. N.L. neut. n. *alkanum*, saturated aliphatic hydrocarbon; L. inf. v. *vorare*, to eat; N.L. part. adj. *alkanivorans*, alkane-devouring).

Gram-negative rod-shaped, aerobic bacterium isolated from a natural gas seep (river water and sediment) located in Pipe Creek, NY, USA. Strain PC2 contains SDIMO-related genes including a putative butane monooxygenase, a toluene monooxygenase, and a phenol hydroxylase. The genome is characterised by a size of 4.0 Mb and has a G + C content of 67.3 mol%.

The type genome of the species has been deposited at the NCBI under the accession number PRJNA752515.

Both isolates are available upon request to the corresponding author.

## Conclusions

In this study, we isolated gaseous-alkane degraders, characterised their monooxygenase enzymes involved in oxidation of ethane, propane, and butane, and retrieved metagenome-assembled genomes from natural gas seeps after DNA-SIP incubations with ^13^C-labelled ethane or propane. We demonstrate the relative abundance and importance of SDIMO family enzymes in the consumption of gaseous-alkanes at these natural gas seep sites. Metagenome data from these SIP experiments have revealed the diversity of active alkane-degraders present and the diversity of SDIMOs they contain and provides vital molecular data for screening and targeted isolation of new and potentially novel ethane and propane-degrading bacteria.

## Supplementary information

Supplementary material

## Data Availability

Sequence data were deposited in the NCBI Sequence Read Archive (SRA) under the bioproject accession numbers: PRJNA765146 for amplicon-sequencing data, PRJNA748243 for metagenomic raw data, and PRJNA748244 for metagenome-assembled genomes. Genomes of the isolates were deposited in the GenBank under the bioproject accession numbers PRJNA752515 (for isolate PC2), PRJNA762293 (for isolate PC3), and PRJNA762315 (for isolate ANDR5).
