# Supplementary material for: Identification of active gaseous-alkane degraders at natural gas seeps
Source: ISME J. 2022 Mar 22;16(7):1705–16. doi: 10.1038/s41396-022-01211-0 (PMC9213486; doi:10.1038/s41396-022-01211-0)
Supplement: Supplementary file 1 — Supplementary material [file 41396_2022_1211_MOESM1_ESM.pdf]

Supplementary information

Identification of active gaseous alkane degraders at natural gas seeps

Muhammad Farhan Ul Haque[1,2*], Marcela Hernández[1,3*‡], Andrew T. Crombie[3] and J. Colin Murrell[1]

[1] School of Environmental Sciences, University of East Anglia, Norwich NR4 7TJ, UK
[2] School of Biological Sciences, University of the Punjab, Quaid-i-Azam Campus, Lahore 54000, Pakistan
[3] School of Biological Sciences, University of East Anglia, Norwich NR4 7TJ, UK

* These authors contributed equally to this work

‡ Corresponding Author

Running title: Natural gas-consuming bacteria in gas seeps

<u>TABLES</u>

Table S1. Summary of comparisons between the isolates characterised in the present study against their closest-relative type strain. Isolates PC2 and PC3 were compared with *Rhodoblastus acidophilus* DSM 137^T (Bioproject PRJNA396223). Isolate ANDR5 was compared against *Mycolicibacterium litorale* F4^T (Bioproject PRJNA374925). All analyses were performed using BLASTn (16S rRNA gene) and BLASTx (for translated sequences of house-keeping genes and SDIMO genes).

Table S2. Percentage identity between MAG19a and isolate ANDR5 for undefined SDIMO (*see Figures 4 and 5*) and *pmoCAB* operons (analysis was performed using BLASTn).

Table S3. Percentage identity of the MAG SDIMO and membrane-bound clusters with sequences in the NCBI database. The table shows the % sequence identity of the translated amino acid sequence of each gene, in comparison with the database entries shown. This table supports data shown in *Figure 5*.

FIGURES

Figure S1. Consumption of ethane (A, B) and propane (C, D) by environmental samples from natural gas seep sites of Pipe Creek (A, C) and Andreiasu Everlasting Fire (B, D). Microcosms containing environmental samples were incubated under ^{13}C-ethane/propane (red circles) or ^{12}C-ethane/propane (blue circles) as the only sources of C or energy without any supplementary nutrients. Independent duplicate incubations were performed for each substrate at each harvested concentration.

Figure S2. DNA retrieved as a function of density of each fraction recovered from density gradient tubes following ultracentrifugation of DNA from SIP incubated samples. Samples from Pipe Creek (A, C) and Andreiasu Everlasting Fire (B, D) were incubated with ethane (A, B) or propane (C, D). DNAs retrieved from heavy and light fractions (encircled).

Figure S3. Community profile of the enriched heavy (C-13 H) and light (C-13 L) DNA fractions of ^{13}C-ethane incubations from DNA-SIP experiment with Pipe Creek samples (A) and Andreiasu Everlasting Fire samples (B), analysed by 16S rRNA gene amplicon sequencing. Profiles of heavy (C-12 H) and light (C-12 L) fractions of control incubations with ^{12}C-ethane are also presented. Taxa present at a relative abundance lower than 1% are included as ‘others’.

Figure S4. Community profile of the enriched heavy (C-13 H) and light (C-13 L) DNA fractions of ^{13}C-propane incubations from DNA-SIP experiment with Pipe Creek samples (A) and Andreiasu Everlasting Fire samples (B), analysed by 16S rRNA gene amplicon sequencing. Sequencing community profiles of heavy (C-12 H) and light (C-12 L) fractions of control incubations with ^{12}C-propane are also presented. Taxa present at a relative abundance lower than 1% are included as ‘others’.

Figure S5. Growth and gas consumption of the isolates PC2 (A, D), PC3 (B, E) and ANDR5 (C, F) on ethane (A, B, C) or propane (D, E, F). Cultures were grown in DNMS medium with ethane/propane (10% v/v) as the only carbon and energy source. Error bars represent the standard error of three (for PC2 and PC3) and two (for ANDR5) replicates.

Figure S6. Phylogenetic tree of the PmoA membrane-bound particulate methane monooxygenase using the Maximum Likelihood method with a JTT matrix-based model. Bootstrap values (100 replications) are shown at the nodes. Values in parenthesis indicate the completeness and contamination of each MAG and genome.

Table S1. Summary of comparisons between the isolates characterised in the present study against their closest-relative type strain. Isolates PC2 and PC3 were compared with *Rhodoblastus acidophilus* DSM 137^{T} (Bioproject PRJNA396223). Isolate ANDR5 was compared against *Mycolicibacterium litorale* F4^{T} (Bioproject PRJNA374925). All analyses were performed using BLASTn (16S rRNA gene) and BLASTx (for translated sequences of house-keeping genes and SDIMO genes).

Gene	PC2	PC3	ANDR5*
16S rRNA	97.6	97.6	97.6
House-keeping genes:			
rpoB	92.6	92.6	95.9
secA	83.1	83.1	89.1
gyrB	87.8	87.8	91.4
rho	94.3	94.3	73.3
murB	81.6	81.6	78.6
era	85.0	85.0	94.0
recR	88.8	88.8	96.0
dapD	84.5	84.5	78.1
aroC	88.9	88.9	86.1
nusG	92.6	92.6	86.4
SDIMO genes:			
bmoX	no hits	no hits	na
prmA	na	na	39.9**

***gyrB, dapD,* and *aroC* were compared against *M. litorale* NIIDNTM18^{T} (Bioproject PRJDB9155) because these genes were not found in *M litorale* F4^{T}
**accession number GenBank: CP019882
na means that the isolates do not possess these genes, therefore no BLASTX was performed
no hits means that the reference type strain does not contain *putative bmoX*

Table S2. Percentage identity between MAG19a and isolate ANDR5 for undefined SDIMO (*see Figures 4 and 5*) and *pmoCAB* operons (analysis was performed using BLASTn)

Query-1	Query-2	gene	Identity (%)
MAG19a	ANDR5	undefined-smoX	100
MAG19a	ANDR5	undefined-smoY	99.83
MAG19a	ANDR5	undefined-smoB	99.61
MAG19a	ANDR5	undefined-unknown	100
MAG19a	ANDR5	undefined-smoC	99.35
MAG19a	ANDR5	undefined-smoZ	97.52
MAG19a	ANDR5	undefined-pmoC	99.87
MAG19a	ANDR5	undefined-pmoA	99.61
MAG19a	ANDR5	undefined-pmoB	99.46

Table S3. Percentage identity of the MAG SDIMO and membrane-bound clusters with sequences in the NCBI database. The table shows the % sequence identity of the translated amino acid sequence of each gene, in comparison with the database entries shown. This table supports data shown in *Figure 5*

SDIMO - Group I

Label	Gene	Identity (%)	Organism	Acc Number	size (aa)
MAG15P	*tmoA*	86.4	*Xanthobacter* sp. 126	WP_029877423.1	498
	tmoB	83.53	*Xanthobacter* sp. 126	WP_024281450.1	86
	tmoC	81.98	*Roseiarcus fermentans*	WP_113890011.1	111
	tmoD	84.62	*Roseiarcus fermentans*	WP_113890010.1	104
	tmoE	80.31	*Roseiarcus fermentans*	WP_113890009.1	328
	tmoF	67.27	*Roseiarcus fermentans*	WP_113890008.1	331
MAG16P	*tmoA*	88.55	*Phyllobacteriaceae* bacterium	NLH83782.1	499
	tmoB	81.18	*Xanthobacter* sp. 126	WP_024281450.1	86
	tmoC	75.68	*Xanthobacter* sp. 126	WP_024280160.1	111
	tmoD	59.78	*Alphaproteobacteria* bacterium	PPR23938.1	101
	tmoE	76.83	*Acidocella aromatica*	WP_183266377.1	328
	tmoF	63.36	*Xanthobacter* sp. 126	WP_024280157.1	333
MAG39P	*tmoA*	93.9	*Roseiarcus fermentans*	WP_113890013.1	500
	tmoB	79.07	*Xanthobacter* sp. 126	WP_024281450.1	86
	tmoC	80.18	*Roseiarcus fermentans*	WP_113890011.1	111
	tmoD	88.46	*Roseiarcus fermentans*	WP_113890010.1	104
	tmoE	82.62	*Roseiarcus fermentans*	WP_113890009.1	328
	tmoF	62.35	*Roseiarcus fermentans*	WP_113890008.1	331
MAG22A	*tmoA*	99.2	Betaproteobacteria bacterium HGW-Betaproteobacteria-4	PKO39569.1	504
	tmoB	95.45	Betaproteobacteria bacterium HGW-Betaproteobacteria-6	PKO36345.1	88
	tmoC	95.5	Betaproteobacteria bacterium HGW-Betaproteobacteria-4	PKO39567.1	111
	tmoD	91.78	Rhodocyclaceae	TXT26377.1	146
	tmoE	95.43	Betaproteobacteria bacterium HGW-Betaproteobacteria-6	PKO36343.1	328
	tmoF	94.38	Rhodocyclaceae bacterium	TXT26379.1	338
MAG26A	*tmoA*	100	*Hydrogenophaga* sp. T4	EWS65867.1	501
	tmoB	100	*Hydrogenophaga* sp. T4	EWS65868.1	88
	tmoC	100	*Hydrogenophaga* sp. T4	EWS65869.1	131
	tmoD	100	*Hydrogenophaga* sp. T4	EWS65870.1	105
	tmoE	100	*Hydrogenophaga* sp. T4	EWS65871.1	328
	tmoF	83.53	Comamonadaceae	TXT41341.1	345
MAG27A	*tmoA*	81.2	*Rugosibacter aromaticivorans*	AJP48258.1	500
	tmoB	67.44	Burkholderiaceae	WP_062085393.1	88
	tmoC	81.08	*Thiomonas delicata*	WP_094162189.1	111
	tmoD	67.74	*Pseudomonas oleovorans*	QCT24444.1	148
	tmoE	79.88	*Thiomonas delicata*	WP_094161766.1	328
	tmoF	69.97	*Zoogloea* sp. 1C4	WP_153160040.1	3380
Isolate PC2	*tmoA*	94.52	*Roseiarcus fermentans*	WP_113890013.1	500
	tmoB	81.4	*Xanthobacter* sp. 126	WP_024281450.1	86
	tmoC	80.18	*Roseiarcus fermentans*	WP_113890011.1	111
	tmoD	85.86	*Xanthobacter* sp. 126	WP_051460639.1	111
	tmoE	84.45	*Roseiarcus fermentans*	WP_113890009.1	328
	tmoF	63.25	*Roseiarcus fermentans*	WP_113890008.1	331
Isolate PC3	*tmoA*	87.98	*Xanthobacter* sp. 126	WP_029877423.1	498
	tmoB	81.4	*Xanthobacter* sp. 126	WP_024281450.1	86
	tmoC	76.58	*Xanthobacter* sp. 126	WP_024280160.1	111
	tmoD	85.86	*Xanthobacter* sp. 126	WP_051460639.1	111
	tmoE	78.96	*Xanthobacter* sp. 126	WP_024280158.1	330
	tmoF	60.36	*Xanthobacter* sp. 126	WP_024280157.1	333

Table S3. continued…

SDIMO - Group II

Label	Gene	Identity (%)	Organism	Acc Number	size (aa)
MAG15P	*dmpK*	67.07	*Roseiarcus fermentans*	WP_113891933.1	97
	dmpL	72.98	*Roseiarcus fermentans*	WP_113894196.1	285
	dmpM	78.89	*Xanthobacter* sp. 126	WP_024280165.1	90
	dmpN	87.23	*Xanthobacter* sp. 126	WP_024280164.1	505
	dmpO	68.03	*Roseiarcus fermentans*	WP_113887731.1	122
	dmpP	78.35	*Roseiarcus fermentans*	WP_113887730.1	352
	dmpQ	71.68	*Methylocella silvestris* BL2	ACK50431.1	117
MAG18P	*dmpK*	100	*Methylocella tundrae*	WP_174512780.1	114
	dmpL	100	*Methylocella tundrae*	WP_174512779.1	336
	dmpM	100	*Methylocella tundrae*	WP_134491646.1	90
	dmpN	100	*Methylocella tundrae*	WP_174512778.1	512
	dmpO	100	*Methylocella tundrae*	WP_174512777.1	119
	dmpP	100	*Methylocella tundrae*	WP_174512776.1	350
	dmpQ	100	*Methylocella tundrae*	WP_174512775.1	122
MAG38P	*dmpK*	85.87	*Rhodoferax* sp. OTU1	WP_114968194.1	92
	dmpL	88.79	*Rhodoferax* sp. OTU1	WP_114968195.1	330
	dmpM	86.52	*Rhodoferax* sp. OTU1	WP_114968196.1	89
	dmpN	94.77	*Rhodoferax* sp. OTU1	WP_114968197.1	515
	dmpO	90.68	*Rhodoferax* sp. OTU1	WP_114968198.1	118
	dmpP	87.96	*Rhodoferax* sp. OTU1	WP_114968199.1	357
	dmpQ	93.81	Comamonadaceae bacterium	TXT36339.1	113
MAG39P_cluster1	*dmpK*	52.33	*Xanthobacter* sp. 126	WP_155982352.1	116
	dmpL	64.55	*Xanthobacter* sp. 126	WP_024280166.1	329
	dmpM	74.44	*Xanthobacter* sp. 126	WP_024280165.1	90
	dmpN	87.3	*Xanthobacter* sp. 126	WP_024280164.1	504
	dmpO	68.42	*Xanthobacter* sp. 126	WP_024280163.1	122
	dmpP	71.35	*Xanthobacter* sp. 126	WP_024280162.1	351
	dmpQ	69.72	*Methylocella silvestris* BL2	WP_113887729.1	117
MAG39P_cluster2	*dmpL*	77.1	*Thauera butanivorans*	WP_068635106.1	330
	dmpM	76.4	*Zavarzinia aquatilis*	WP_109907407.1	89
	dmpN	85.77	*Zavarzinia aquatilis*	WP_109907406.1	506
	dmpO	65.25	*Zavarzinia compransoris*	WP_109919211.1	118
	dmpP	71.67	*Ponticaulis* sp.	MAI91238.1	353
MAG22A_cluster1	*dmpL**	92.02	*Dechloromonas* sp. SG708	WP_168658543.1	330
	dmpM	98.88	*Dechloromonas* sp. TW-R-39-2	WP_203468262.1	89
	*dmpN**	96.3	*Dechloromonas hortensis*	WP_153130320.1	514
MAG22A_cluster2	*dmpL**	77.78	*Dechloromonas aromatica*	WP_011289521.1	329
	dmpM	84.27	*Dechloromonas* sp. HYN0024	WP_117609873.1	89
	*dmpN**	90.16	*Dechloromonas* sp. CZR5	WP_150427615.1	518
MAG22A_cluster3	*dmpN**	88.17	*Dechloromonas* sp.	KAB2917872.1	518
	*dmpO**	89.92	Azonexaceae bacterium	NTV72394.1	119
MAG26A	*dmpK*	100	*Hydrogenophaga* sp. T4	EWS65901.1	71
	dmpL	100	*Hydrogenophaga* sp. T4	EWS65902.1	331
	dmpM	100	*Hydrogenophaga* sp. T4	EWS65903.1	89
	dmpN	82.29	*Rhodoferax* sp. OTU1	WP_114969329.1	519
	dmpO	100	*Hydrogenophaga* sp. T4	EWS65905.1	118
	dmpP	86.44	*Rhodoferax* sp. OTU1	WP_114969327.1	354
	dmpQ	87.72	*Rhodoferax* sp. OTU1	WP_114969326.1	115
MAG27A	*dmpK*	59.62	*Hydrogenophaga crassostreae*	WP_066085342.1	69
	dmpL	76.97	*Rhodoferax* sp. OTU1	WP_114969331.1	331
	dmpM	80.9	*Hydrogenophaga* sp. T4	EWS64923.1	89
	dmpN	88.39	*Leptothrix cholodnii*	WP_012348365.1	528
	dmpO	85.59	*Hydrogenophaga* sp. T4	EWS65905.1	118
	*dmpP**	81.64	*Rhodoferax* sp. OTU1	WP_161124060.1	354
Isolate PC2_cluster	*dmpK*	63.01	Phyllobacteriaceae bacterium	NLH81109.1	97
	dmpL	73.03	*Azospirillum* sp. TSO22-1	WP_183267306.1	330
	dmpM	78.89	*Roseiarcus fermentans*	WP_113894195.1	90
	dmpN	97	*Xanthobacter* sp. 126	WP_024280164.1	504
	dmpO	57.14	*Martelella radicis*	WP_183481446.1	123
	dmpP	70.11	*Xanthobacter* sp. 126	WP_024280162.1	351
	dmpQ	52.38	*Phenylobacterium* sp.	MBJ7411950.1	301
Isolate PC2_cluster	*dmpK*	67.11	Phyllobacteriaceae bacterium	NLH81109.1	97
	dmpL	69.34	*Roseiarcus fermentans*	WP_113894196.1	285
	dmpM	74.44	*Xanthobacter* sp. 126	WP_024280165.1	90
	dmpN	88.71	*Xanthobacter* sp. 126	WP_024280164.1	504
	dmpO	70.25	*Roseiarcus fermentans*	WP_113892168.1	122
	dmpP	70.2	*Xanthobacter* sp. 126	WP_024280162.1	351
	dmpQ	71.05	*Methylocella silvestris* BL2	ACK50431.1	117
Isolate PC3_cluster	*dmpN**	88.64	*Xanthobacter* sp. 126	WP_024280164.1	504
	dmpO	70.25	*Roseiarcus fermentans*	WP_113892168.1	122
	*dmoP**	88.46	*Roseiarcus fermentans*	WP_113887730.1	352
Isolate PC3_cluster	*dmpN**	81.36	*Xanthobacter* sp. 126	WP_024280164.1	504
	dmpO	56.67	*Roseiarcus fermentans*	WP_113887731.1	122
	*dmoP**	81.4	*Roseiarcus fermentans*	WP_113887730.1	352
Isolate PC3_cluster	*dmpL**	60.54	*Azospirillum* sp. TSO22-1	WP_109118499.1	333
	dmpM	78.89	*Rhodopila globiformis*	WP_104519433.1	90
	*dmpN**	91.49	*Xanthobacter* sp. 126	WP_024280164.1	504
Isolate PC3_cluster	*dmpP**	78.36	*Roseiarcus fermentans*	WP_113887730.1	352

* contig trimmed

Table S3. continued…

SDIMO - Group III

Label	Gene	Identity (%)	Organism	Acc Number	size (aa)
MAG12A	*mmoX*	96.39	*Methylobacter* sp.	TAN65936.1	527
	mmoY	92.86	*Methylobacter* sp.	TAN65935.1	392
	mmoB	92.14	*Methylobacter* sp.	TAN65934.1	140
	mmoZ	84.43	*Methylobacter* sp.	TAN65933.1	168
	mmoD	75.38	*Methylobacter* sp.	PPD43327.1	83
	mmoC	86.67	*Methylobacter* sp.	TAN65931.1	345
	unknown function	57.58	*Methylobacter tundripaludum*	WP_104425273.1	101
	mmoG	78.25	*Methylobacter* sp.	PPD43324.1	562
MAG16P	*bmoX*	99.24	*Methylocella tundrae*	VTZ27606.1	528
	bmoY	98.28	*Methylocella tundrae*	VTZ27607.1	406
	bmoB	97.01	*Methylocella tundrae*	WP_174513143.1	134
	bmoZ	97.35	*Methylocella tundrae*	VTZ27609.1	189
	bmoD	88.31	*Methylocella tundrae*	WP_174513141.1	77
	bmoC	99.1	*Methylocella tundrae*	VTZ27611.1	346
	unknown function	97.83	*Methylocella tundrae*	VTZ27612.1	118
	bmoG	98.7	*Methylocella tundrae*	VTZ27817.1	539
MAG32A	*bmoX*	94.89	*Methylocella tundrae*	VTZ27606.1	528
	bmoY	82.02	*Methylocella tundrae*	VTZ27607.1	406
	bmoB	89.55	*Methylocella tundrae*	WP_174513143.1	134
	bmoZ	75.13	*Methylocella tundrae*	VTZ27609.1	189
	bmoD	65.62	*Methylocella tundrae*	WP_174513141.1	77
	bmoC	75.43	*Methylocella tundrae*	WP_174513140.1	364
	unknown function	71.76	*Methylocella tundrae*	VTZ27612.1	118
	bmoG	79.21	*Methylocella tundrae*	VTZ28270.1	547
Isolate PC2	*bmoX*	86.96	*Azoarcus* sp. DD4	WP_141018055.1	531
	bmoY	75.19	*Azoarcus* sp. DD4	WP_141018054.1	395
	bmoB	78.36	*Azoarcus* sp. DD4	WP_141018053.1	137
	bmoZ	71.69	*Azoarcus* sp. DD4	WP_141018052.1	168
	bmoC	68.87	*Azoarcus* sp. DD4	WP_141018048.1	363
	unknown function	59.34	*Azoarcus* sp. DD4	WP_141018047.1	105
	bmoG	55.81	*Azoarcus* sp. DD4	WP_141018046.1	567
Isolate PC3	*bmoX*	86.96	*Azoarcus* sp. DD4	WP_141018055.1	395
	bmoY	75.19	*Azoarcus* sp. DD4	WP_141018054.1	395
	bmoB	76.87	*Azoarcus* sp. DD4	WP_141018053.1	138
	bmoZ	71.69	*Azoarcus* sp. DD4	WP_141018052.1	168
	bmoC	68.87	*Azoarcus* sp. DD4	WP_141018048.1	363
	unknown function	59.34	*Azoarcus* sp. DD4	WP_141018047.1	105
	bmoG	55.3	*Azoarcus* sp. DD4	WP_141018046.1	567

Table S3. continued…

SDIMO - Group V

Label	Gene	Identity (%)	Organism	Acc Number	size (aa)
MAG2P	*prmA*	95	*Mycolicibacterium moriokaense*	WP_114737585.1	542
	prmB	87.69	*Mycobacterium* sp. JS623	WP_015305468.1	347
	prmC	86.98	*Mycobacterium* sp. JS623	WP_015305467.1	366
	prmD	92.73	*Mycobacterium* sp. SWH-M5	WP_073678483.1	114
	prmG	88.16	*Mycobacterium* sp. 852013-50091_SCH5140682	WP_064946231.1	548
MAG17P	*prmA*	98.19	*Methylocella tundrae*	WP_134492912.1	552
	*prmB***	98.21	*Methylocella tundrae*	WP_134492910.1	362
MAG18P	*prmA*	100	*Methylocella tundrae*	WP_134492912.1	552
	prmB	97.51	*Methylocella tundrae*	VTZ25485.1	362
	prmC	81.79	*Methylocella silvestris*	WP_102841856.1	357
	prmD	99.15	*Methylocella tundrae*	VFU16488.1	118
	prmG	100	*Methylocella tundrae*	WP_174513727.1	547
	prmR	100	*Methylocella tundrae*	WP_174513728.1	693
MAG47P	*prmA*	95.86	*Rhodobacter sphaeroides* *	*WP_002720019.1	555
	prmB	87.04	*Amaricoccus* sp. HB172011	WP_140454575.1	358
	prmC	86.61	*Ensifer aridi*	WP_028002705.1	352
	prmD	87.8	*Mesorhizobium*	WP_073989699.1	122
	a	88.24	*Mesorhizobium*	WP_126039948.1	340
	b	66.4	*Amaricoccus* sp. HB172011	WP_140454661.1	258
	prmG	87.45	*Amaricoccus macauensis*	WP_184149405.1	542
	prmR	70.95	*Amaricoccus* sp. HB172011	WP_140454662.1	649
MAG49P	*prmA*	92.95	Rhodobacteraceae bacterium HLUCCA08	KPP93819.1	554
	prmB	80.63	Rhodobacteraceae bacterium HLUCCA08	KPP93820.1	351
	prmC	84.42	Rhodobacteraceae	WP_009572587.1	353
	prmD	86.89	*Rhodobacter*	WP_002720016.1	122
MAG33A	*prmA*	98.74	*Rhodomicrobium*	WP_088348746.1	554
	prmB	93.98	*Rhodomicrobium*	WP_088348745.1	349
	prmC	96.71	*Rhodomicrobium*	WP_088348744.1	365
	prmD	99.16	*Rhodomicrobium*	WP_088348806.1	119
	a	95.81	*Rhodomicrobium*	WP_088348743.1	334
	b	88.05	*Rhodomicrobium*	WP_088348742.1	255
	prmG	93.39	*Rhodomicrobium*	WP_088348741.1	545

* In 2020 it was recommended that *Rhodobacter sphaeroides* be moved to the genus Cereibacter. This is the name currently used by the NCBI taxonomy database.

** contig trimmed

a: amidohydrolase

b: iron-sulfur cluster assembly protein

Table S3. continued…

SDIMO - Group VI

Label	Gene	Identity (%)	Organism	Acc Number	size (aa)
MAG19A_cluster 1	*prmA*	95.13	*Mycobacterium* sp. ENV421	WP_102810306.1	533
	prmC	88.71	*Mycolicibacterium fluoranthenivorans*	WP_187099574.1	363
	prmD	98.86	*Mycolicibacterium chubuense* NBB4	ACZ56326.1	89
	prmB	87.46	*Mycobacterium dioxanotrophicus*	WP_087083749.1	343
MAG19A_cluster 2	*prmA*	95.64	*Mycobacterium* sp. TY-6	BAF34294.1	516
	prmC	94.02	*Mycobacterium* sp. TY-6	BAF34295.1	351
	prmD	89.91	*Mycobacterium* sp. TY-6	BAF34296.1	110
	prmB	89.87	*Mycobacterium* sp. TY-6	BAF34297.1	316
Isolate ANDR5_cluster 1	*prmA*	95.48	*Mycolicibacterium*	WP_014805366.1	512
	prmC	88.71	*Mycolicibacterium fluoranthenivorans*	WP_187099574.1	363
	prmD	98.86	*Mycolicibacterium chubuense* NBB4	ACZ56326.1	89
	prmB	87.17	*Mycobacterium dioxanotrophicus*	WP_087083749.1	343
Isolate ANDR5_cluster 2	*prmA*	95.05	*Mycobacterium* sp. TY-6	BAF34294.1	517
	prmC	94.02	*Mycobacterium* sp. TY-6	BAF34295.1	351
	prmD	89.91	*Mycobacterium* sp. TY-6	BAF34296.1	110
	prmB	89.87	*Mycobacterium* sp. TY-6	BAF34297.1	316

Table S3. continued…

SDIMO - Undefined group

Label	Gene	Identity (%)	Organism	Acc Number	size (aa)
MAG19A	*smoX*	87.76	*Mycolicibacterium chubuense* NBB4	ACZ56334.1	531
	smoY	72.36	*Mycobacterium*	WP_014805751.1	399
	smoB	75.15	*Mycolicibacterium*	WP_014805750.1	175
	unknown function	69.35	*Mycolicibacterium rhodesiae* NBB3	AEV73592.1	62
	smoC	87.15	*Mycolicibacterium fluoranthenivorans*	WP_187099622.1	347
	smoZ	70.59	*Mycolicibacterium*	WP_014805748.1	188
Isolate ANDR5	*smoX*	87.76	*Mycolicibacterium chubuense* NBB4	ACZ56334.1	531
	smoY	72.11	*Mycobacterium*	WP_014805751.1	399
	smoB	77.64	*Mycolicibacterium rhodesiae*	WP_014211364.1	173
	unknown function	69.35	*Mycolicibacterium rhodesiae* NBB3	AEV73592.1	62
	smoC	88.18	*Mycolicibacterium rhodesiae*	WP_014211366.1	347
	smoZ	75.4	*Mycolicibacterium rhodesiae*	WP_014211367.1	188

Table S3. continued…

Membrane-bound

Label	Gene	Identity (%)	Organism	Acc Number	size (aa)
MAG2P	*pmoC*	67.65	*Mycobacterium* sp. ENV421	WP_102810152.1	292
	pmoA	78.52	*Mycolicibacterium chubuense* NBB4	ADT71671.1	276
	pmoB	62.83	*Mycobacterium* sp. ENV421	WP_102810154.1	425
MAG14P	*pmoA**	83.28	*Methylocapsa acidiphila*	CAJ01617.1	295
	pmoB	78.17	*Methylocapsa aurea*	WP_051953405.1	426
MAG19A	*pmoC*	77.97	*Mycobacterium* sp. ENV421	WP_102810152.1	292
	pmoA	78.21	*Mycolicibacterium chubuense* NBB4	ADT71671.1	276
	pmoB	66.91	*Mycobacterium* sp. ENV421	WP_102810154.1	425
MAG32A	*pmoC*	71.49	Rhizobiales	RTL80976.1	265
	pmoA	70.23	Rhizobiales	RTL80975.1	271
	pmoB	56.8	Rhizobiales	RTL80974.1	347
Isolate ANDR5	*pmoC*	73.88	*Mycobacterium* sp. ENV421	WP_102810152.1	292
	pmoA	78.21	*Mycolicibacterium chubuense* NBB4	ADT71671.1	276
	pmoB	66.91	*Mycobacterium* sp. ENV421	WP_102810154.1	425

* contig trimmed

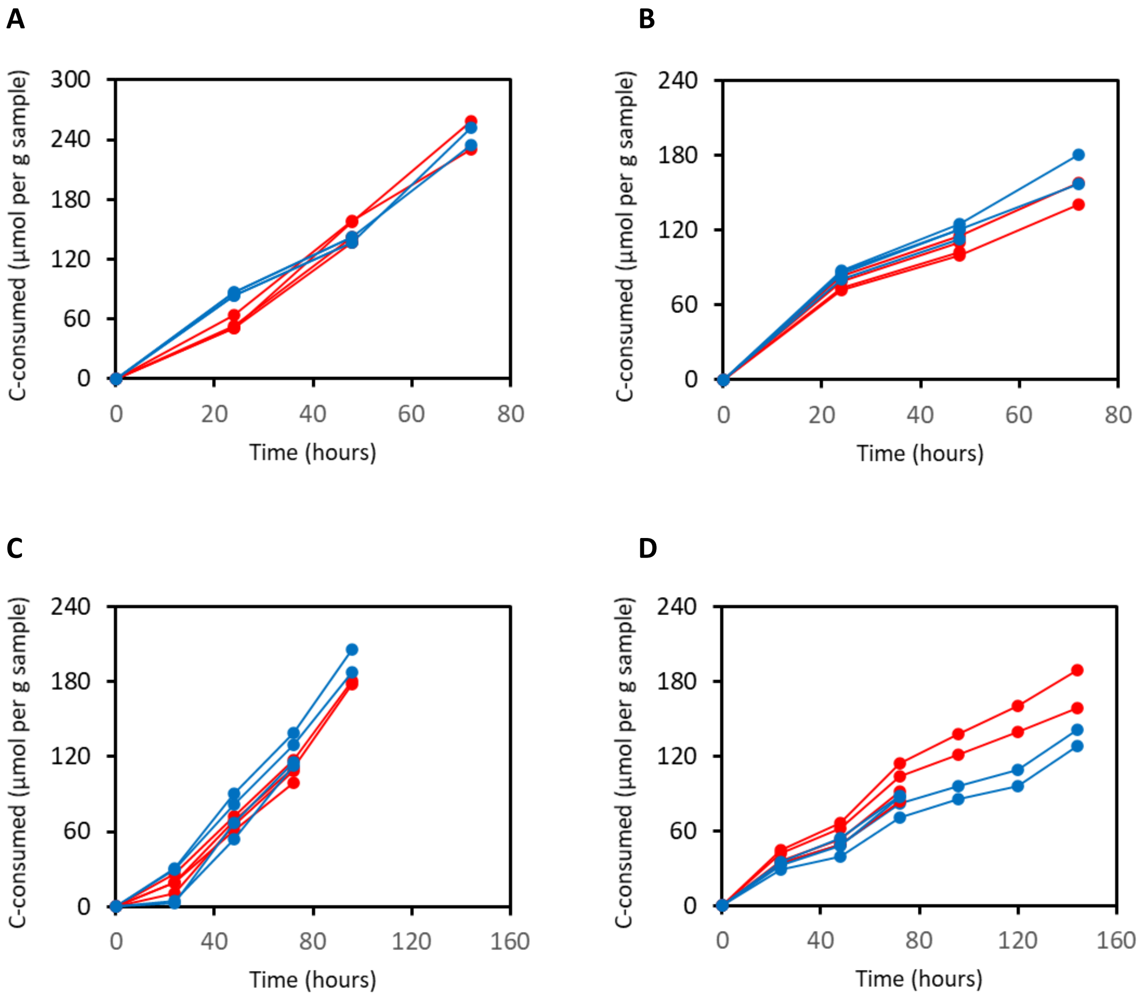

Figure S1. Consumption of ethane (A, B) and propane (C, D) by environmental samples from natural gas seep sites of Pipe Creek (A, C) and Andreiasu Everlasting Fire (B, D). Microcosms containing environmental samples were incubated under ^{13}C-ethane/propane (red circles) or ^{12}C-ethane/propane (blue circles) as the only sources of C or energy without any supplementary nutrients. Independent duplicate incubations were performed for each substrate at each harvested concentration.

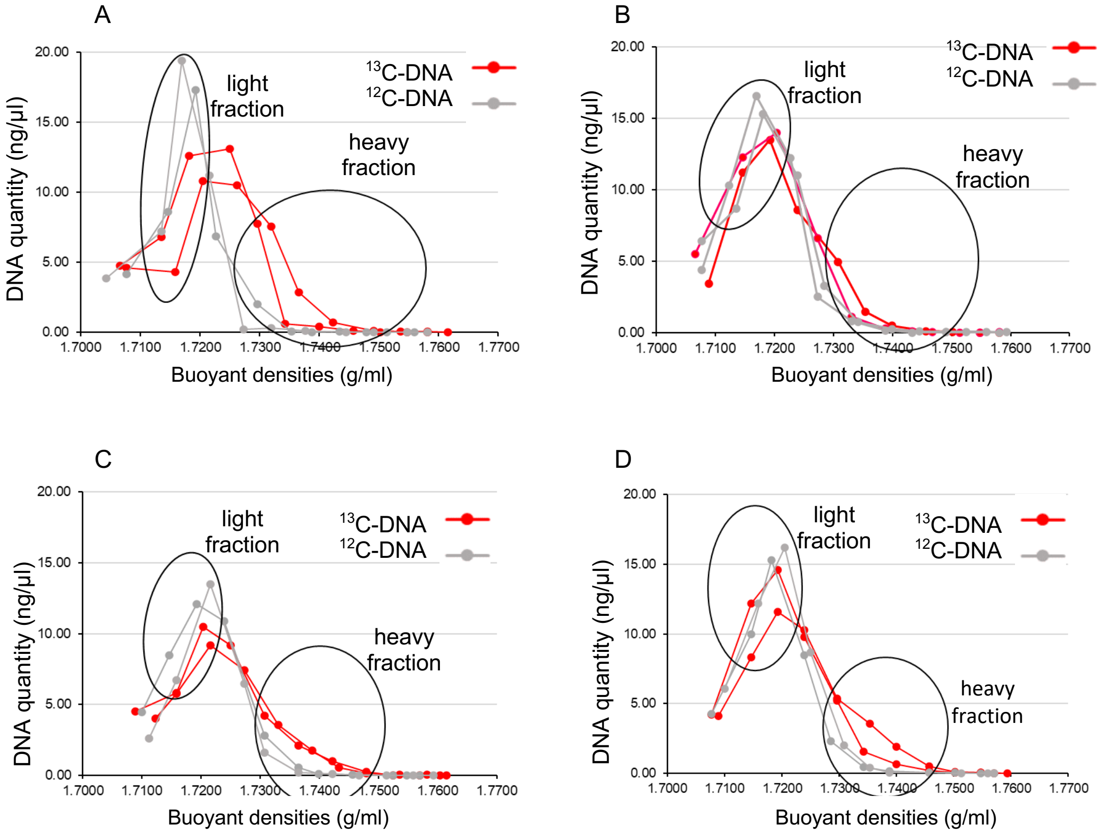

Figure S2. DNA retrieved as a function of density of each fraction recovered from density gradient tubes following ultracentrifugation of DNA from SIP incubated samples. Samples from Pipe Creek (A, C) and Andreiasu Everlasting Fire (B, D) were incubated with ethane (A, B) or propane (C, D). DNAs retrieved from heavy and light fractions (encircled).

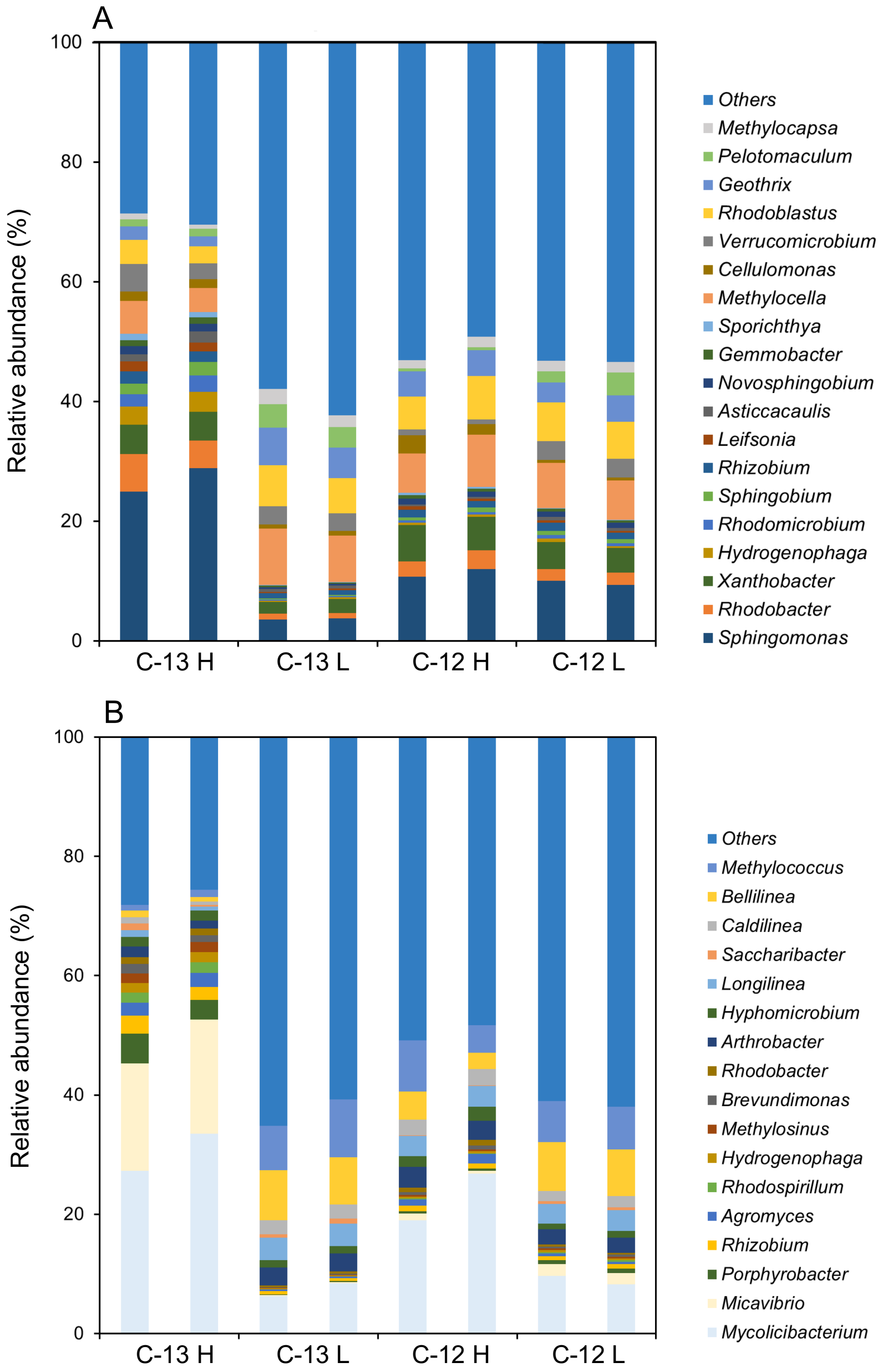

Figure S3. Community profile of the enriched heavy (C-13 H) and light (C-13 L) DNA fractions of ^{13}C-ethane incubations from DNA-SIP experiment with Pipe Creek samples (A) and Andreiasu Everlasting Fire samples (B), analysed by 16S rRNA gene amplicon sequencing. Profiles of heavy (C-12 H) and light (C-12 L) fractions of control incubations with ^{12}C-ethane are also presented. Taxa present at a relative abundance lower than 1% are included as ‘others’.

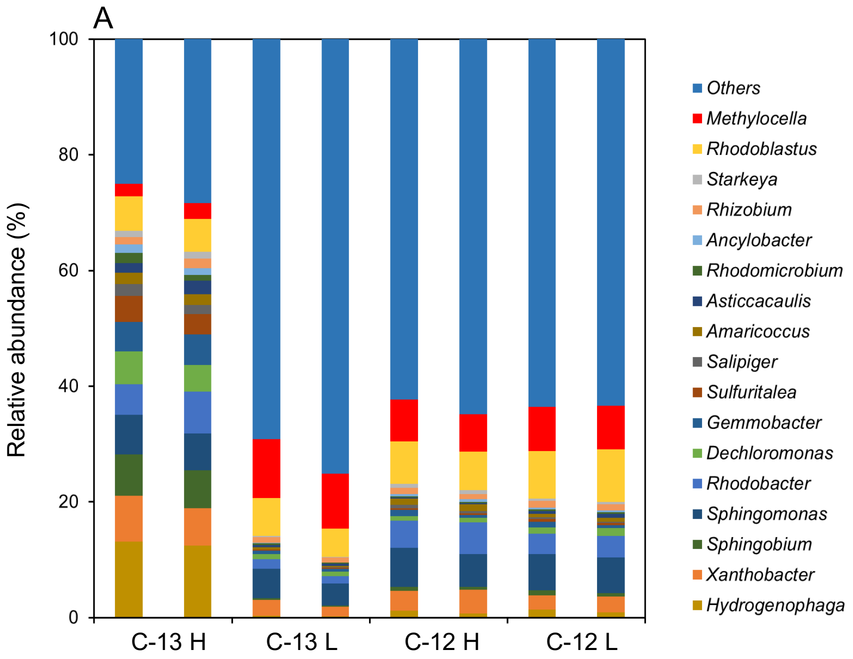

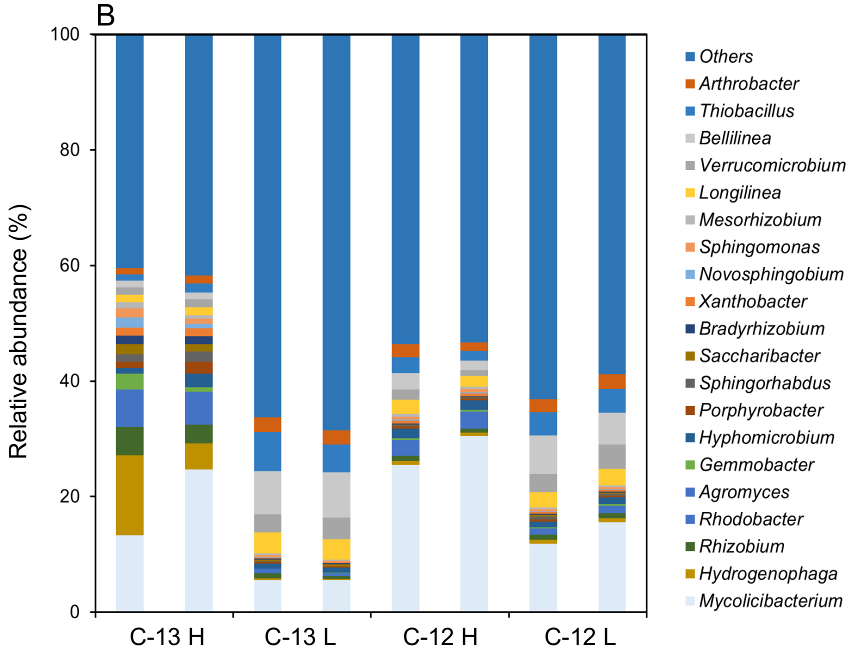

Figure S4. Community profile of the enriched heavy (C-13 H) and light (C-13 L) DNA fractions of ^{13}C-propane incubations from DNA-SIP experiment with Pipe Creek samples (A) and Andreiasu Everlasting Fire samples (B), analysed by 16S rRNA gene amplicon sequencing. Sequencing community profiles of heavy (C-12 H) and light (C-12 L) fractions of control incubations with ^{12}C-propane are also presented. Taxa present at a relative abundance lower than 1% are included as 'others'.

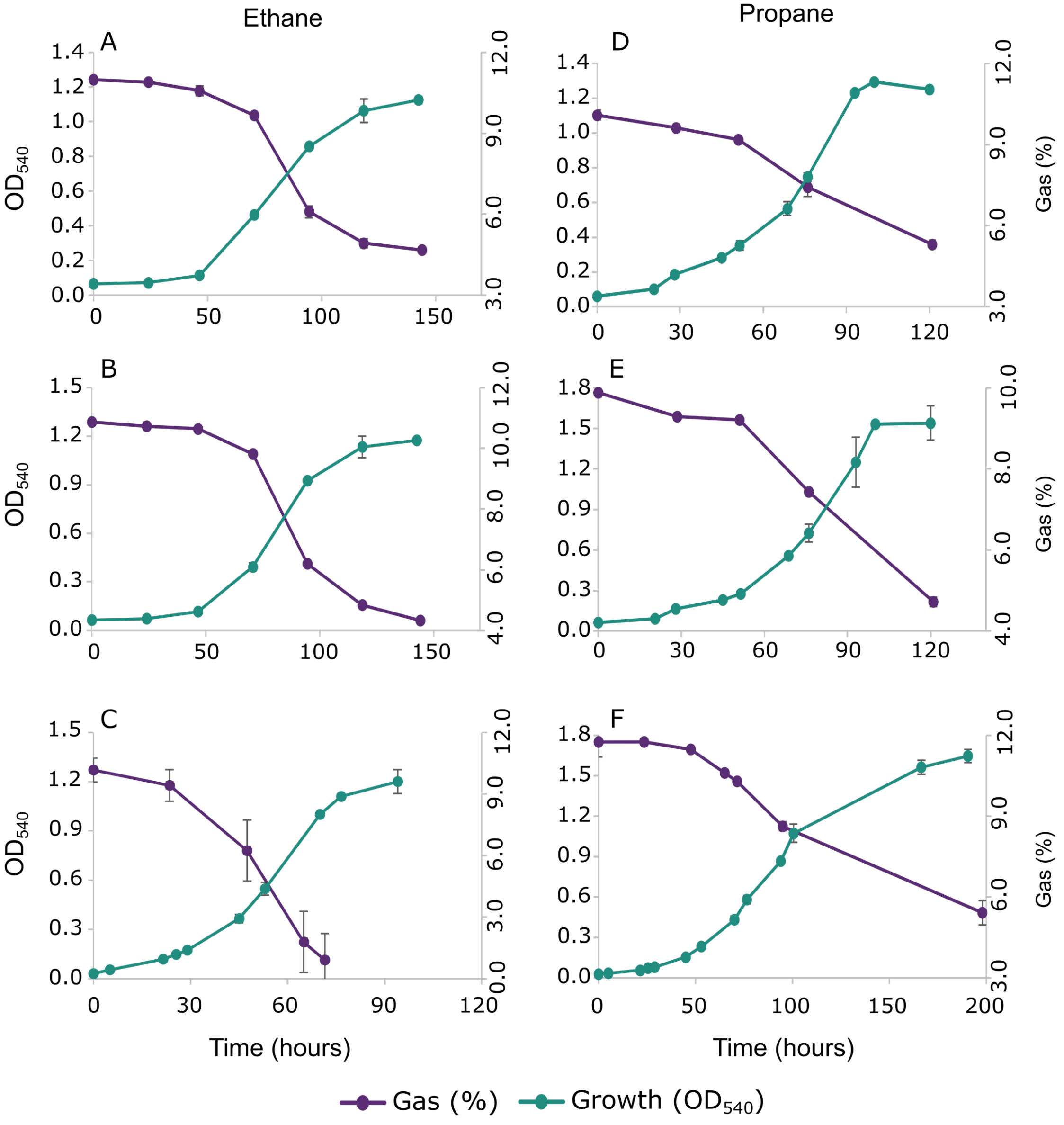

Figure S5. Growth and gas consumption of the isolates PC2 (A, D), PC3 (B, E) and ANDR5 (C, F) on ethane (A, B, C) or propane (D, E, F). Cultures were grown in DNMS medium with ethane/propane (10% v/v) as the only carbon and energy source. Error bars represent the standard error of three (for PC2 and PC3) and two (for ANDR5) replicates.

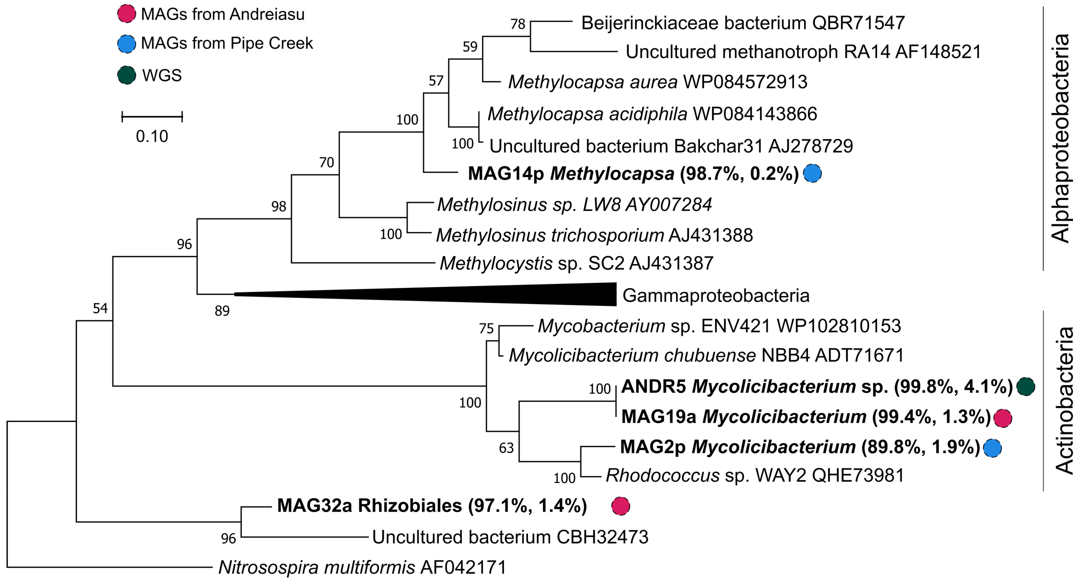

Figure S6. Phylogenetic tree of the PmoA membrane-bound particulate methane monooxygenase using the Maximum Likelihood method with a JTT matrix-based model. Bootstrap values (100 replications) are shown at the nodes. Values in parenthesis indicate the completeness and contamination of each MAG and genome.
